# Comprehensive Immunoglobulin G, A, and M Glycopeptide Profiling for Large-Scale Biomedical Research

**DOI:** 10.1016/j.mcpro.2025.100928

**Published:** 2025-02-19

**Authors:** Bianca D.M. van Tol, Anna M. Wasynczuk, Steinar Gijze, Oleg A. Mayboroda, Jan Nouta, Radboud J.E.M. Dolhain, Manfred Wuhrer, David Falck

**Affiliations:** 1 Center for Proteomics and Metabolomics, Leiden University Medical Center, Leiden, the Netherlands; 2 Department of Rheumatology, Erasmus University Medical Center, Rotterdam, The Netherlands

**Keywords:** antibody glycosylation, glycopeptides, immunoglobulin, liquid chromatography, mass spectrometry, relative quantification, rheumatoid arthritis, site-specific glycan analysis

## Abstract

Glycosylation of immunoglobulin G (IgG) is recognized as a key modulator of cellular effector functions. At the same time, an increasing body of evidence underlines the importance of other antibody isotypes, especially IgA and IgM, in pathophysiological conditions. Therefore, methods to efficiently study the complex interplay between isotypes, subclasses, and glycosylation of antibodies during acute and chronic states of inflammation are needed. As a solution, we present an integrated and comprehensive method combining simultaneous affinity enrichment of IgG, IgA, and IgM with a single measurement, glycopeptide-centered LC-MS analysis of all isotypes which provides protein-specific (isotype and subclass), and site-specific N- and O-glycosylation quantitation. A two-protease approach provided individual peptides for each glycosylation site, allowing unambiguous compositional assignment and relative quantitation of glycoforms on the MS^1^ level as well as structural confirmation and partial isomer assignment on the MS/MS level. We demonstrate that our methodology can be efficiently applied to large clinical studies revealing differences in antibody glycosylation in women during and after pregnancy, as well as between healthy donors and patients with rheumatoid arthritis. In addition, this showcased the advantages of our method in comprehensiveness and resolution of isotypes, subclasses, and glycosylation sites as well as its precision and robustness.

## Highlights

• We developed an integrated method for IgG, IgA, and IgM glycoanalysis by LC-MS.
• N- and O-glycosylation is analyzed isotype-, subclass-, and site-specifically.
• Large sample cohorts can be efficiently assessed (>100 per week).
• IgG, IgA, and IgM, glycosylation changes with pregnancy and rheumatoid arthritis.
• IgG/A/M glycosylation is associated with these conditions in shared and divergent ways.

## Graphical abstract

**Figure:**
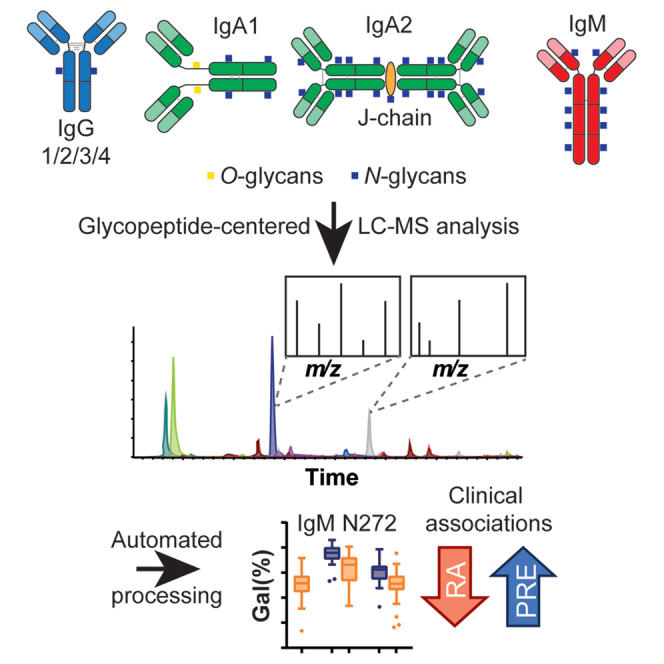

## In Brief

This article describes an integrated and comprehensive method for the analysis of IgG, A, and M glycosylation by LC-MS. By a glycopeptide-based approach, it efficiently quantifies N- and O-glycosylation in an isotype-, subclass-, and site-specific manner for hundreds of samples in a matter of weeks. To demonstrate the feasibility of a large-scale analysis and the power of the method, we recapitulated and extended associations of IgG, A, and M glycosylation with pregnancy and rheumatoid arthritis in a cohort of 172 samples.

Glycosylation modulates the structure and receptor recognition of antibodies (1). Antibody glycosylation is a key determinant of their effector functions and has been associated with various homeostatic and pathogenic changes (2, 3). IgG Fc N-glycosylation varies with sex, age, and ethnicity, and differs for different target antigens (2, 4). Assessment of antibody glycosylation furthers our understanding of immunological processes and may result in clinical markers (5). Functional roles for immunoglobulin G (IgG) glycosylation (4, 6) and IgA O-glycosylation (7, 8) are proven, and roles of IgA N-glycosylation are emerging (9, 10). For example, afucosylation of IgG is a key determinant of Fc gamma receptor-mediated antibody-dependent cellular cytotoxicity (4), while IgA1 O-glycans deficient in galactose are targets for autoantibodies in IgA nephropathy (7, 8). However, there is little knowledge about the roles of IgM glycosylation (11), partially due to the lack of efficient methods to analyze IgM glycosylation in depth in a site-specific manner. An integrated method for all three isotypes, IgG, IgA, and IgM, is needed to efficiently study the known roles of one isotype while discovering new roles for the others.

**Fig. 1:**
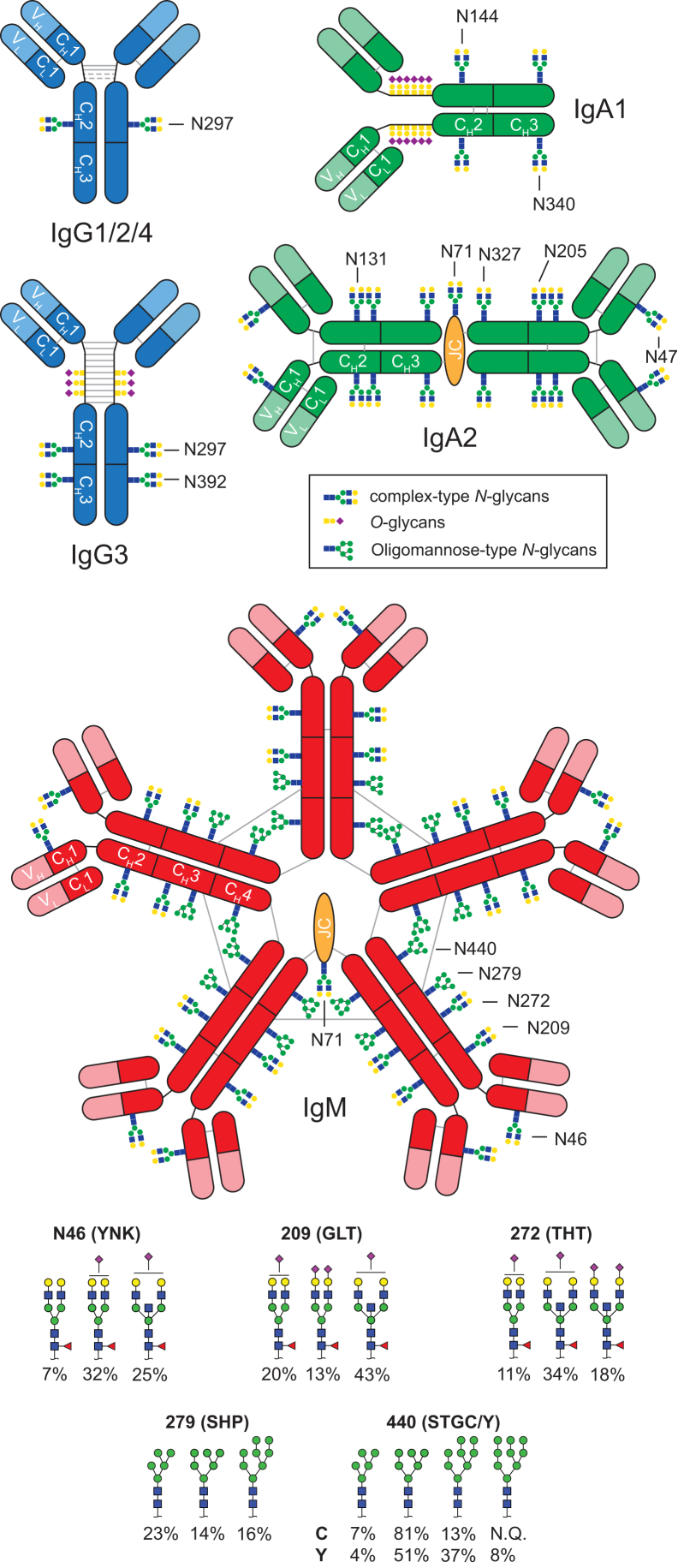
**Antibody glycosylation.** Schematic representation of the glycosylation sites of IgG1/2/3/4 (monomers), IgA1 (monomer), IgA2 (dimer), and IgM (pentamer) on constant domains, numbered according to literature (2). Each Ig monomer consists of constant domains (*Dark blue* = IgG, *dark green* = IgA, and *dark red* = IgM) and variable regions (*Light blue*, *green*, and *red*). IgA2 and IgM are shown in their multimeric form in complex with the joining chain (JC; *orange*). JC is the same for IgA2 and IgM and also contains a glycosylation site (N71).

During the primary adaptive immune response, B-cells produce immunoglobulin M (IgM) as the first response to antigens. Since affinity maturation has not occurred yet, IgMs have a low affinity for the antigens. However, IgM is secreted as covalently attached pentamers or hexamers which increases avidity. The pentamer contains, in addition, a joining chain (JC) (Fig. 1). Throughout the immune response, antibodies may undergo affinity maturation. Then, a class switch to the production of IgG and IgA may occur, in which the constant region of the antibody heavy chain is changed, but the variable region stays intact, preserving the antigen specificity. IgM is the third most common Ig in the circulation (appr. 0.4–2.3 mg/ml) after IgG and IgA (typically 7–16 mg/ml and 0.7–4.0 mg/ml) (12). As opposed to the four and two subclasses of IgG and IgA, respectively, IgM features a single constant domain sequence. However, allotypes of IgM have been reported (P01871 IGHM_HUMAN, https://www.uniprot.org/uniprotkb/P01871/entry, (13)).

IgM caries many co- and post-translational modifications (PTMs). An ubiquitous and multifaceted PTM is glycosylation which is indirectly encoded in the genome and heavily influenced by environmental factors. IgM has five conserved N-glycosylation sites with varying macro- and microheterogeneity. Asn 46, Asn 209, and Asn 272 mainly contain complex-type glycans, while the CH3 domain sites Asn 279 and Asn 440 mainly contain oligomannosidic glycans. Hybrid-type glycans were previously detected on Asn 46, Asn 209, and Asn 279 (Fig. 1) (14, 15). The joining chain, identical in IgA and IgM, also has one N-glycosylation site.

IgM glycosylation has been studied using released glycan strategies (16). However, site-specific glycosylation information is essential for furthering our structure-function understanding of IgM. Thus, different approaches for analyzing IgM glycosylation on the glycopeptide level have been developed, for example, using nanoLC-microarray-MALDI-MS (15), nanoLC-MS/MS (17, 18), or LC-MS (14).

Many immune responses are complex and involve various antibody classes which may display specific glycosylation changes. Therefore, a comprehensive method for glycosylation analysis of all three major isotypes provides an unprecedented opportunity to efficiently study antibody-dependent immunity. Recently, a workflow based on a selected reaction monitoring (SRM) approach was published which covered in total 95 glycopeptides of IgG, IgA, and IgM (19). While the number of transitions covered is impressive, SRM relies on internal stable-isotope labeled standards for every analyte to compensate for the low MS resolution and the differences in fragmentation efficiency. Yet, the study only used four peptide standards which led to a lack of accuracy for several glycosylation features, especially sialylation. Potentially due to the cost factor associated with the internal standard, a limited number of samples was analyzed. In contrast, our workflow can adapt to new samples, bringing new glycoforms, to the data processing level, without necessitating additional method development. This results in much higher coverage of glycoforms and a focus on important differences when comparing different samples.

**Fig. 2:**
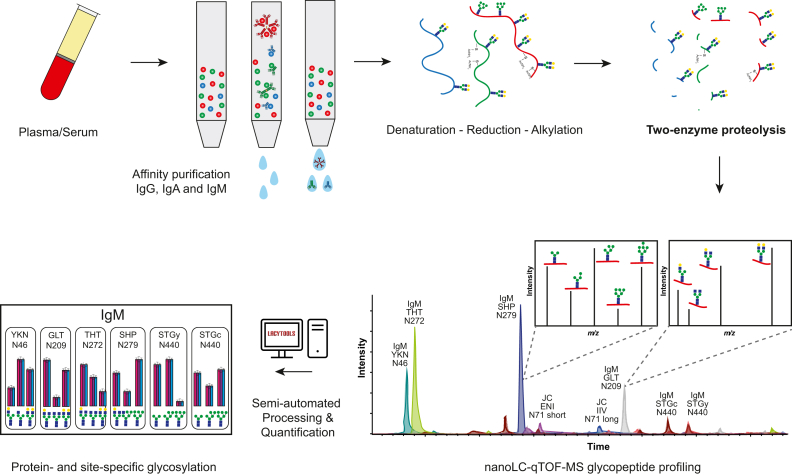
**Schematic overview of the workflow for antibody glycosylation profiling.** The total pool of IgG, IgA, and IgM antibodies from serum or plasma is enriched using affinity purification. The antibodies are acid denatured, disulfide bonds are reduced and free thiols are alkylated. Unfolded polypeptide chains are cleaved by trypsin and endoGluC into a pool of proteolytic (glyco-)peptides. These glycopeptides are analyzed by nanoLC-qTOF-MS with separation primarily based on the peptide backbone. The different glycopeptides present in every cluster are quantified semi-automatically using LaCyTools and GlycoDash.

We here present a workflow for the sensitive and comprehensive analysis of IgM, IgA, and IgG glycosylation from large sets of plasma samples (Fig. 2). The method provides a comprehensive glycoproteomics overview of antibody immunity from minute amounts of serum or plasma. This is based on principles employed earlier in a combined IgG and IgA glycosylation analysis workflow (20).

The method has a well-match computational workflow for data processing which ensures sufficient throughput from sample to relative glycan abundances. We demonstrated this on a set of 160 clinical samples from the Pregnancy-induced Amelioration of Rheumatoid Arthritis (PARA) cohort where the glycosylation pattern (pre-), during, and post-pregnancy were compared between healthy women and women with rheumatoid arthritis (RA).

## Experimental Procedures

### Samples

Serum samples from 36 RA patients and 32 healthy volunteers (without adverse obstetric histories) were obtained from the PARA study (21). Characteristics of patients and healthy controls have been described previously and are given in Table 1 (22). In brief, the age and duration of pregnancy were the same between cases and controls. Samples from participants were obtained in the third trimester of the pregnancy and 26 weeks postpartum. Only RA patients donated an additional sample preconception. All pregnancies were completed, and all patients fulfilled the 1987 American College of Rheumatology (ACR) criteria for RA. Samples were collected between 2002 and 2009. This study was approved by the Medical Ethics Committee at the Erasmus MC, University Medical Center Rotterdam, Rotterdam, The Netherlands, and complies with the Declaration of Helsinki.

**Table 1:** Participant characteristics for the evaluated PARA study subset

Participant characteristic	Healthy volunteers (n = 32)	RA patients (n = 36)
Age at delivery	32.1 (4.4)	32.5 (4.0)
Pregnancy duration in weeks	40.1 (1.4)	39.2 (1.9)
Disease duration at first visit in years	-	7.3 (5.8)
ACPA positive patients, n (%)	-	22 (61%)
Rheumatoid factor positive patients, n (%)	-	25 (69%)
Erosive disease, n (%)	-	14 (39%)
Disease activity score (DAS23 (3)-CRP)) at preconception	-	3.8 (1.0)

Values are extracted from literature (22) and represent mean and, in brackets, standard deviation unless otherwise indicated.

All patients were seen and bled at their home address throughout the Netherlands. Blood samples were stored at room temperature and frozen within 24 h. Short-term storage (days to a few weeks) at −20 °C was followed by long-term storage at −80 °C. Serum samples were stored in 2 ml aliquots, were further aliquoted to 100 μl upon first use, and stored at −80 °C. We showed previously that IgG glycosylation is very stable under long-term storage even with repeated freeze-thaw cycles (23).

Immunoglobulin M (Lee Biosolutions, #340–30), Immunoglobulin A (Athens Research & Technology, #16–16–090701), Immunoglobulin G (Athens, #16–16–090707), and VisuCon-F Frozen Normal Control Plasma (Stago BNL, #FRNCP0125) were used as a technical standards. Negative PBS controls consisting of 1× PBS (32 mM disodium hydrogen phosphate (Sigma-Aldrich, cat. no. 71643), 3.5 mM potassium dihydrogen phosphate (Merck, cat. no. 1.04873) and 145 mM sodium chloride (Sigma-Aldrich, cat. no. 31434); pH 7.6.), pH 7.6, made in-house from 5.7 g Na_2_HPO_4_·2 H_2_O/L, 0.476 g KH_2_PO_4_/L and 8.5 g NaCl/L in water). were included at random locations in the sample plate layout (*N* = 4). For other chemicals, see supplementary methods.

### Experimental Design and Statistical Rational

We selected three time points for 36 patients with RA and two time points of 32 healthy volunteers, in total 172 samples. These samples were mainly selected to allow a comparison with previous investigations (22). The post-partum time point served as clinical control for the pregnancy effect, and the two time points of the healthy volunteers as clinical controls for RA and mixed effects. Visucon-F pooled plasma standard (10 replicates) was used as a full technical control. We regularly use this standard in our investigation which allows us to compare the method performance with previous studies. All samples and standards were arranged in random order over two 96-well plates for sample preparation and measurement.

Glycosylation traits reflecting structural motifs as well as biosynthetic steps were calculated as described previously to strengthen statistical analysis and facilitate biological and clinical interpretation, see supplemental Table S3 (24). For example, the glycosylation trait IgG1 galactosylation, reflection caping of antennae in IgG1 complex glycans with galactoses, is calculated by summing the relative intensities of the glycans containing galactose, counting those diantennary structures with two galactoses full and those with one half. The linear mixed-effects models were analyzed using the R lme4 package (version 1.1–35.4). We built individual models for each glycosylation trait, with trait levels used as the outcome variable. The fixed effects included group, time point, and their interaction (group ∗ time_point). Random intercepts were included for patients to account for repeated measures within individuals. We interpreted results with −2<t > 2 as findings and with an estimate >0.5 as strong findings. Multiple testing corrections using the Bonferroni method yielded an adjusted α of 0.0006. Again, the statistical approach largely reflected the choices made in a previous investigation of the same dataset which we aimed to replicate and extend (22).

### Affinity Enrichment of Immunoglobulins from Serum or Plasma

Five microlitre of serum or plasma was pipetted into 96-well PCR plates (Greiner Bio-One B.V., #652270) containing 120 μl PBS. IgM was isolated from serum or plasma similarly to Momcilovic *et al*. (20) IgM capturing bead suspension (2 × 900 μl) was spun for 10 min @ 100 *g* and the supernatant (2 × 450 μl) was removed. Beads were resuspended in 900 μl of 20% aqueous EtOH and spun for 10 min @ 100 *g*. The supernatant was removed (2 × 450 μl) and beads were resuspended in PBS (2 × 3006 μl), yielding 2 × 3456 μl bead slurry (containing 450 μl beads). 30 μl of IgM bead slurry (containing 3.9 μl IgM beads; theoretical capacity 19.5 μg IgM), was applied to 96-well filter plates (10 μm pore size, Orochem) and beads were washed three times with 200 μl PBS using a vacuum manifold (50 kPa pressure gradient). 30 μl PBS was added to each well to prevent drying. 125 μl of PBS-prediluted serum or plasma (5 μl serum or plasma) was transferred to each filter plate well. Beads were incubated for 1h at room temperature with shaking at 1050 rpm with a 1.5 mm orbit. After incubation, the beads were dried and washed three times with 200 μl PBS and three times with 200 μl water, using a vacuum manifold except for spinning for 5 min @ 100 *g* in the last step. For elution, 100 μl of 100 mM formic acid was added, followed by 5 min shaking as above. Eluates were collected into nonskirted 96-well PCR plates (Greiner, #652201) by 2 × 5 min centrifugation at 100*g* and dried by vacuum centrifugation for 2.5 h at 50 °C. IgG, IgA, and IgM were isolated simultaneously from serum according to the same protocol, using 30 μl of IgM bead slurry (3.9 μl IgM beads), and 40 μl of IgG + IgA bead slurry (0.04 μl IgG beads with 1 μg IgG capacity and 5 μl IgA beads with 40 μg IgA capacity).

### Glycopeptide Preparation

Dried eluates were dissolved in 10 μl of reduction-alkylation buffer (50 mM ABC, 10 mM TCEP, and 40 mM CAA), sealed with VersiCap Mat, Flat Cap Strips (Thermo Fisher Scientific), and shaken for 5 min at 450 rpm with 1.5 mm orbit. Denaturation, reduction, and alkylation is performed in a single step by incubation for 5 min at 95 °C in a 2720 Thermal Cycler (Thermo). After mixing with 15 μl of 50 mM ABC for 5 min at 300 rpm with a 1.5 mm orbit, 25 μl of the protease solution was added. The protease solution contained 15 μl of trypsin solution and 15 μl of Glu-C solution in 270 μl ice-cold water (10 ng/μl Trypsin and 10 ng/μl Glu-C). Sequencing-grade Trypsin (100 μg) was dissolved in ice-cold 20 mM HAc (100 μl) and sequencing-grade Glu-C (50 μg) in ice-cold water (50 μl). The plate was closed with VersiCap Mat, and Flat Cap Strips and incubated overnight at 37 °C. Afterward, the plate was stored at −20 °C before MS analysis.

### Glycopeptide Analysis by Liquid Chromatography-Mass Spectrometry

Thawed, mixed, and spun (5 min at 1200 *g*) protease digests were analyzed by nano-liquid chromatography (LC) - electrospray ionization (ESI) - mass spectrometry (MS) as described previously (20). An Ultimate 3000 RSLCnano system (Dionex/Thermo Fisher Scientific, Sunnyvale, CA) coupled to an Impact quadrupole time-of-flight-MS (Bruker Daltonics, Bremen, Germany) was used. Deviations from the previous method are limited to a 200 nl injection volume, 0.02% aqueous TFA as solvent A, and a shorter run time (21–17 min), specifically a shorter re-equilibration time (8–4 min) (20). The LC-MS data, including fragmentation data, have been deposited to the ProteomeXchange Consortium via the PRIDE partner repository (25) with the dataset identifier PXD057528.

### Data Processing

Raw LC-MS data was processed according to a recently published protocol (26), using the following software versions: Proteowizard 3.0 suite (Version 3.0.23037) and LaCyTools version 2.1.0 build 20,230,525 (https://git.lumc.nl/cpm/lacytools/-/releases) (27). A list of 547 potential analytes was prepared from literature and manual inspection of spectra (supplemental Table S1). Targeted, spectral calibration and integration used the settings listed in supplemental Tables S1 and S2 (27). The LaCyTools output was curated using GlycoDash, an R Shiny dashboard for spectral and analyte curation, glycosylation trait calculation, and metadata integration (https://github.com/Center-for-Proteomics-and-Metabolomics/GlycoDash). Data curation was based on isotopic pattern quality score (IPQ <0.2), signal-to-noise ratio (S/N > 9), and absolute mass error (<20 ppm) and is described in detail in the supplementary methods. Quantitation within each glycosylation side was based on elemental compositions, meaning glycan isomers were not distinguished.

### Confirmation of Glycopeptide Identity by Fragmentations Techniques

IgM (P01871–1) glycopeptides were generated as described above and subjected to LC-MS/MS analysis on an Orbitrap Fusion LUMOS mass spectrometer similar to previous studies (28). In short, MS1 full scan spectra *m/z* 300 to 2000 and MS/MS stepping energy (25, 32, and 39% NCE), high-energy collision-induced dissociation spectra were acquired. Manual annotation of spectra was supported by output from Byonic (Protein Metrics, Cupertino, CA, v5.1.1). The Byonic search was performed using unspecific cleavage, 10 ppm precursor mass tolerance, 20 ppm fragment mass tolerance, fixed carbamidomethyl modification, and oxidation, N-terminal acetylation, and N-glycans as variable modifications. The following, generally accepted assumptions were made regarding structures presented in the figures and text: All N-glycans should have the pentasaccharide core; the third and fourth HexNAc are interpreted as GlcNAc on the alpha1-3 and alpha1-6 arm, respectively; non-core hexoses are galactoses extending the arms, unless the number of hexoses exceeds that of HexNAc by two, in which cases non-complex structures are proposed. IgA O-glycans were interpreted as core structures in line with the literature. All other indications of structures, such as bisecting GlcNAc, core fucosylation or that sialic acids are linked to galactose, were derived from the interpretation of the LC-MS/MS data.

## Results

### IgG, IgA, and IgM Enrichment and glycopeptide Generation

IgG, IgA, and IgM were simultaneously enriched from serum or plasma using affinity beads. However, in contrast to IgG and IgA beads, IgM beads suffered from non-specific binding under previously reported conditions (20). This is likely due to the differences in solid support, the IgG and IgA beads being agarose-based while the IgM beads are poly(styrene-divinylbenzene) based. Increasing the sodium chloride concentration in the first three wash steps to 1.14 M resulted in greatly decreased non-specific binding and sufficient purity of the affinity eluate for further processing (supplemental Fig. S1).

Proteolytic cleavage, with trypsin and GluC, combined, resulted in well-defined glycopeptides allowing the individual assessment of all N-glycosylation sites presented in Figure 1. Most sites were observed as a single peptide sequence for all glycopeptides (Table 2) with negligible amounts of glycopeptides of alternative (additional or additional missed) cleavages. No or only minimal oxidation of methionine (sulfoxide or sulfone) was observed. IgM glycopeptides contained 7 to 22 amino acids and all provided good signal intensity. The glycopeptides of the C-terminal glycosylation site N440 showed the expected heterogeneity regarding the presence of the C-terminal tyrosine (17). In IgA, we observed two significant peptide backbone versions for the shared IgA1 and two sites N144/131, due to moderate GluC cleavage efficiency after E142/129. Due to an efficient cleavage after E347/334, a single peptide backbone was obtained for the C-terminal tyrosine variants of IgA. For IgG glycopeptides, the tryptic cleavages were dominant with only minor impurities of cleavages after the glutamic acids at −3 and −4 of the N297 glycosylation site. The joining chain, derived from IgA dimer and IgM pentamer, respectively, showed the two previously reported glycopeptide cleavage variants, resulting from inefficient trypsin cleavage after arginine at −2 from the glycosylation site (20). Only for IgM_N440, a non-glycosylated peptide was observed as both C-terminal tyrosine variants. These observations are in line with previous investigations of antibody site occupancies, showing either a lack of non-glycosylated peptide detection or site occupancies above 99% (17).

**Table 2:** Overview of peptide sequences obtained by combined trypsin and GluC cleavage

Protein	Glycosylation site	Peptide sequence^a^	Cluster^b^	Retention time (sec)	MC	Glyco-forms
IgG1	N297	(R)EEQY**N**STYR(V)	IgG1	51–61	2	17
IgG2/3	N297	(R)EEQF**N**STFR(V)	IgG2/3	133–142	2	7
IgG4	N297	(R)EEQF**N**STYR(V)	IgG4	90–99	2	4
IgA1	O-glycans^c^	(K)HYTNPSQDVTVPCPVPS**T**PP**T**P**S**P**ST**PP**T**PSPSCCHPR(L)	HYT	175–204	-	42
IgA1/A2	N144/131	(E)A**N**LTCTLTGLR(D)	ANL	225–233	-	8
IgA1/A2	N144/131	(E)DLLLGSEA**N**LTCTLTGLR(D)	DLL	347–356	1	5
IgA1/A2	N340/327	(R)LAGKPTHV**N**VSVVMAE(V)	LAGe	206–217		13
IgA2	N47	(W)SESGQ**N**VTAR(N)	SES	26–34	1	7
IgA2	N205	(K)TPLTA**N**ITK(S)	TPL	136–145	-	7
JC	N71	(R)E**N**ISDPTSPLR(T)	ENI	149–161	1	8
JC	N71	(R)IIVPLNNRE**N**ISDPTSPLR(T)	IIV	208–215	2	NQ
IgM	N46	(K)YK**N**NSDISSTR(G)	YKN	20–40	1	30
IgM	N209	(R)GLTFQQ**N**ASSMCVPDQDTAIR(V)	GLT	230–240	-	9
IgM	N272	(K)THT**N**ISE(S)	THT	34–50	-	16
IgM	N279	(E)SHP**N**ATFSAVGE(A)	SHP	134–146	-	14
IgM	N440	(K)STGKPTLY**N**VSLVMSDTAGTC	STGc	262–280	-	3
IgM	N440	(K)STGKPTLY**N**VSLVMSDTAGTCY	STGy	289–300	-	4

MC, Missed cleavages; NQ, not quantified.

a Bold marked Asn, Ser, and Thr are glycosylation sites. The N-glycosylation consensus motif is underlined.

b Names for IgG glycosylation sites refer to their subclass, while the names for the IgA, IgM, and JC refer to the first three letters of the peptide sequence of the glycopeptides.

c T106/T109/S111/S113/T114/T117.

Trypsin cleavage alone resulted in the peptide backbones previously described for IgG and IgA glycopeptides (supplemental Table S4) (20). For IgM, sites N46, N209, and N440 resulted in the same peptide backbones as for the two-protease cleavage (Table 2 and supplemental Table S4). IgM sites N272 and N279 were detected on the same peptide backbone, thus not allowing an individual assessment of these two glycosylation sites when using only trypsin. IgA glycopeptides showed a single backbone for N144/131. Trypsin also allowed the detection of the IgA C-terminal tyrosine variants.

The six potential O-glycosylation sites in the hinge region of IgA were covered by a single, identical peptide backbone, regardless of whether cleavage is enacted with trypsin alone or in combination with GluC.

### Site-Specific IgM Glycosylation

**Fig. 3:**
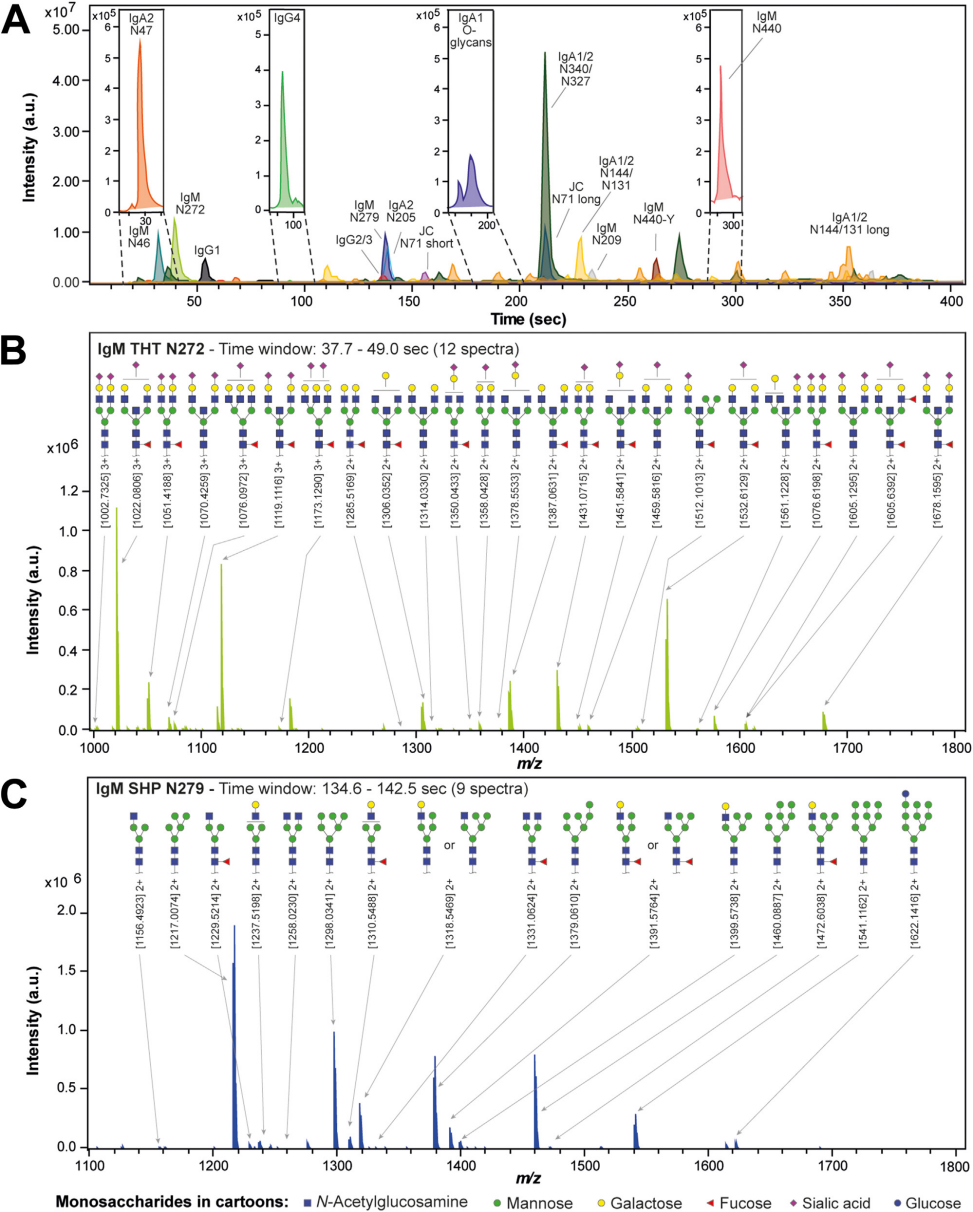
**NanoLC-qTOF-MS glycopeptide profiling of IgG, IgA, and IgM combined.***A*, extracted ion chromatograms (EICs) for the most abundant glycopeptide of each glycosylation site for all covered antibody isotypes. Insets show zooms of glycosylation sites of low intensity. Glycopeptides with the same peptide sequence, but different glycan structures, elute at similar retention times in glycopeptide clusters. For the glycopeptide clusters covering glycosylation sites IgM_N272 (*B*) and IgM_N279 (*C*), sum mass spectra are provided covering all corresponding glycopeptides with the same peptide sequence. Retention time ranges for sum spectra for all glycosylation sites can be found in Table 2.

When applying the two-protease cleavage (Fig. 2), all resulting IgM glycopeptides were well separated per glycosylation site in the chromatographic dimension (Figs. 3*A* and supplemental Fig. S2*A*). N46 and N272 sites were detected with short and/or hydrophilic peptide backbones, limiting the retention of the reversed-phase material. In our experience with the equally early eluting IgA_N47 glycopeptides, it is important to follow the loading step conditions tightly to avoid the loss of these early eluting glycopeptides. All glycosylation sites were detected with high-quality spectra, as exemplified on the IgM_N272 and IgM_N279 sites in Figure 3, *B* and *C*. IgM_N272 showed complex-type glycans with a high degree of bisection, galactosylation, and sialylation. Minor compositions indicating triantennary glycans were also detected. IgM_N279 showed largely oligomannosidic glycans accompanied by a significant portion of hybrid-type glycans and minor amounts of complex-type glycans. Also with the glycopeptides of IgG, IgA, and IgM combined, most were well separated in the chromatographic dimension (Fig. 3*A*). A critical coelution existed between the glycopeptides of IgG2/3, IgM_N279, and IgA2_N205. The long J chain IIV glycopeptides and the IgA1/2_N340/N327 glycopeptides coeluted as well. However, in all cases of coelution, it was possible to unambiguously assign the signal of the mass spectrometric dimension to a glycosylation site and the corresponding glycoform, as exemplified in supplemental Fig. S2*B*.

IgM_N46 and IgM_N209 showed mainly fully galactosylated diantennary glycans which were core fucosylated, partially bisected, and partially sialylated (Fig. 1 and supplemental Fig. S3). Sialylation was higher in IgM_N209 compared to N46. IgM_N440 showed exclusively oligomannose glycans in both C-terminal tyrosine variants. Interestingly, glycoforms of the variant retaining the tyrosine presented larger oligomannoses. The joining chain showed fully galactosylated diantennary glycoforms with a high degree of sialylation and some fucosylation and bisection on its JC_N71 site. Annotations of IgM glycopeptide were confirmed by fragmentation analysis for the two most abundant species per site (supplemental Fig. S4).

### Method Performance

The intermediate precision of the method is excellent (supplemental Fig. S3). Most major analytes show relative standard deviations (RSDs) ≤5% over the cause of the cohort measurement in plasma standard (supplemental Tables S5 and S6). The only notably increased RSDs are found in the glycoforms of the low abundant J-chain and the very early eluting IgM_N46 and IgA_N47 glycopeptides, the latter likely due to the aforementioned issues with trap column retention. In contrast to the low technical variation, biological variation is much larger even within the groups of the PARA cohort. For example, RSDs of the post-partum time point of the healthy volunteer and the RA patient groups are all above 5%, going up to 40% in individual analytes (supplemental Fig. S3 and supplemental Table S6).

The excellent precision was achieved despite a large coverage of, in total, 194 glycopeptides. This allowed monitoring of interesting features, which were not covered by an alternative approach (19), such as afucosylation and oligomannose glycans on IgA1/2 N340/327, JC hybrid type glycans, IgM N46 oligomannose, triantennary and complex agalactosylated glycans, IgM N 205 triantennary glycans, IgM N272 triantennary glycans, and IgM N279 monoantennary glycans and greatly improved oligomannose coverage. Importantly, we could cover IgA1 O-glycosylation, N-glycosylation site IgA2 N47, and site occupancy of IgM N440 in addition.

### Clinical Associations

**Fig. 4:**
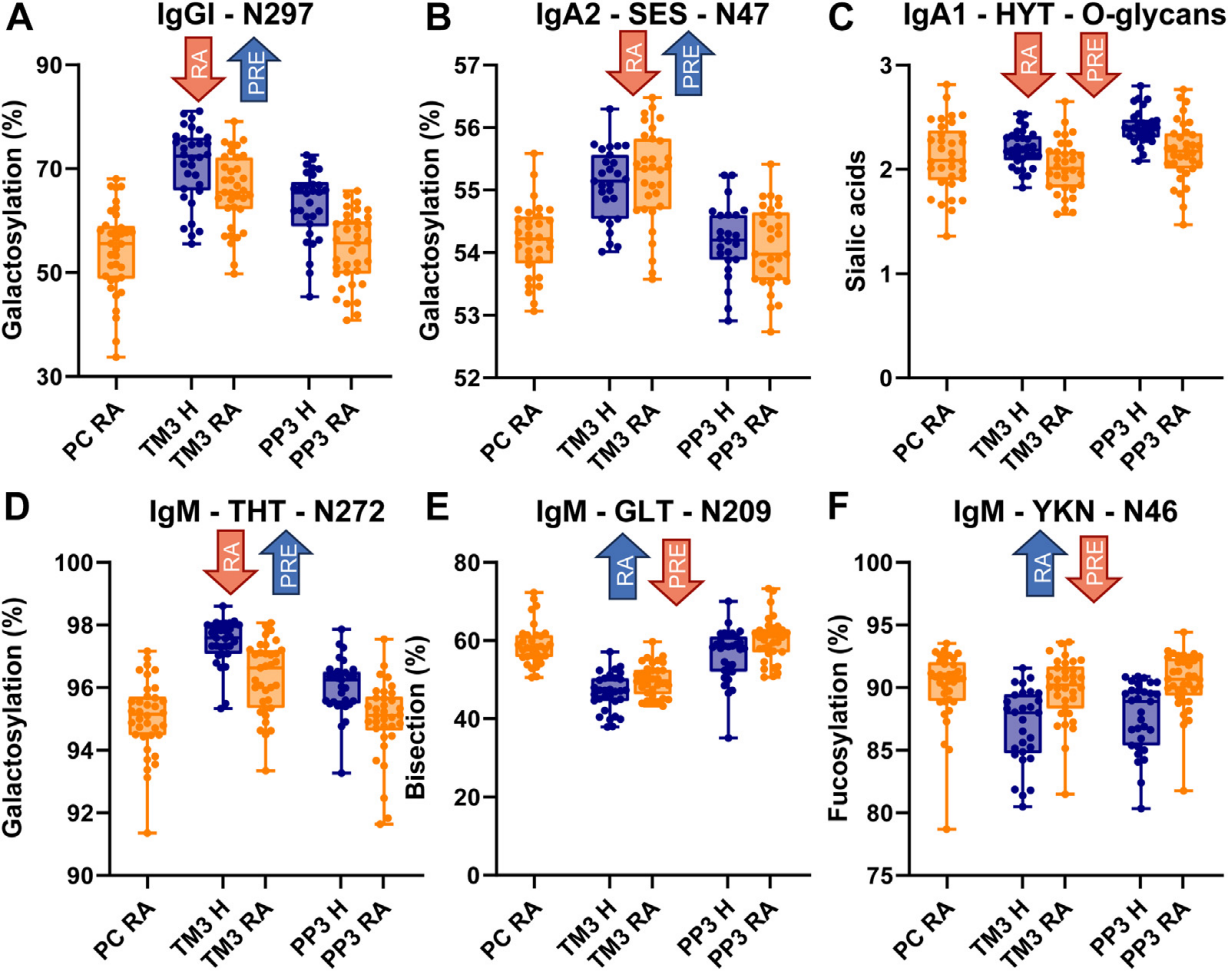
**Isotype-, subclass- and site-specific glycosylation changes on IgG, IgA, and IgM during pregnancy (PRE) and associations with rheumatoid arthritis (RA).** Each panel represents a glycosylation trait (y-axis label, see also supplemental Table S3) for one specific glycosylation site (panel header). Panels (*A*), (*B*), and (*D*) show the relative abundance of galactosylation per antenna of complex type glycan for IgG N297, IgA N47, and IgM N272 sites, respectively, (*E*) summed relative abundance of bisected glycans on IgM N209 and (*F*) summed relative abundance of fucosylated glycans on IgM N46 normalized to the total abundance of complex type glycans. Panel (*C*) shows the average number of sialic acids on O-glycans per IgA1 molecule. Box-and-whisker plots for selected glycosylation traits are shown pre-conception (PC; RA patients only), at the third trimester (TM3), and 3 months post-partum (PP3). Boxes represent medians and interquartile ranges with whiskers representing the full range. *Blue* “up” arrows indicate positive associations, while *red* ‘down’ arrows indicate negative associations. The direction of correlation is derived from the sign of the output values of the model (“estimate” or t-statistics values in supplemental Table S7). The chosen models use both time points (TM3+PP3) to determine differences between healthy volunteers and patients with RA, and both groups to determine differences between time points.

The multivariate mixed linear regression model revealed Ig glycosylation features that were associated with pregnancy as well as with rheumatoid arthritis, including some interactions thereof (supplemental Table S7 and Fig. 4). Instead of individual glycoforms, we used glycosylation traits reflecting biosynthetic steps and structural motifs as further explained in the methods section (see supplemental Table S3 for detailed calculations). The pregnancy time point showed elevated levels of IgG1, IgG2, and IgG4 galactosylation, as reported before without subclass resolution (22), as well as independently of galactosylation increase in IgG sialylation. IgM glycosylation sites largely reflected these associations. IgA N-glycan sialylation was also positively associated with pregnancy except the IgA2 Fab site N47 when corrected for galactosylation changes. IgA galactosylation was only associated positively with pregnancy on IgA2_N47 and IgA1/2_N340/327. In contrast to N-glycosylation, sialylation of IgA O-glycans is associated strongly negatively with pregnancy. In line with previous findings, minding our isotype resolution (22), IgG and IgM bisection decreased during pregnancy (3). IgA bisection was largely unaffected. Core fucosylation of IgG1, IgA1/2_N340/327, IgM_N46, and IgM_N279 was lower in pregnancy, whilst antennary fucosylation appeared to be increased in IgM_N46. Associations of glycan complexity with pregnancy were diverse and varied largely per glycosylation site. All elaborations of joining chain glycosylation are strongly, and negatively associated with pregnancy.

RA is associated with decreased IgG1, 2, and four galactosylation, but not independently with sialylation, as previously reported without subclass resolution (22). IgA and IgM N-glycan galactosylation are also associated negatively with RA. Again, IgA O-glycosylation behaved opposingly from N-galactosylation, with O-glycan galactosylation and sialylation associating strongly positively and negatively with RA, respectively, when corrected for site occupancy. Bisection was strongly positively associated with RA in IgA and IgM, but not in IgG, which is also in line with previous reports (22). Interestingly, IgA2_N205 and IgM_N46 formed exceptions. IgG1 and IgM core fucosylation were increased in RA, but IgA did not. In contrast, IgM antennary fucosylation was negatively associated with RA. Glycan complexity showed little association with RA. Notably, however, IgA O-glycan site occupancy was strongly negatively correlated with RA. Joining chain glycosylation did not associate with RA.

Changes in a few glycosylation features during pregnancy were observed to depend on the RA status (interaction term supplemental Table S7). IgG1 galactosylation increased more with pregnancy in RA patients than in healthy controls. Antennary fucosylation of IgM, but not of IgA, increased less with pregnancy in RA patients. IgA2_N47 sialylation increased more with pregnancy in RA patients compared to healthy volunteers. The higher abundance of the Man5 species on IgM_N46 in RA was less pronounced during pregnancy.

## Discussion

### Technical Improvements and Considerations

Our method combines several key analytical features achieving a simultaneous IgG, IgA, and IgM glycosylation analysis for the sensitive and comprehensive analysis of large sets of plasma samples (Fig. 2). Separating the different peptide clusters with LC, gains sensitivity and avoids overlapping masses between clusters. A nanoESI interface enhanced with dopant-enriched nitrogen gas confers supreme sensitivity and sufficient glycopeptide selectivity to obtain a deep coverage of microheterogeneity without the need for intricate glycopeptide enrichment. The 96-well plate-based sample preparation and short LC gradient allow for a throughput-optimized method able to cover 100s of samples in a matter of days. Finally, automated computational processing makes data available for biological or clinical interpretation in an equally time-efficient manner. All this is achieved from low microliter amounts of serum or plasma.

The relative amount of IgM affinity beads was calculated based on their capacity to achieve similar concentrations for IgG, IgA, and IgM during sample preparation and measurement (20). GluC is required in addition to trypsin to achieve an individual assessment of each glycosylation site for IgM (Table 2 and supplemental Table S4). Specifically, ambiguity exists between compositional GlcNAcs belonging to bisected structures on N272 or hybrid-type structures on N279, as well as between hexoses being galactoses on N272 or mannoses on N279 (Fig. 3, *B* and *C*). In an application focusing on IgM, this makes the two-protease cleavage preferable. However, the IgG and especially the IgA data are slightly simpler and thus more robust in the trypsin-only cleavage (supplemental Table S4). Importantly, C-terminal tyrosine cleavage on IgA can only be assessed in the trypsin-only method.

### IgG/A/M Glycosylation Profiles

LC-MS allowed unambiguous assignment and individual quantitation of glycan sites and compositions. Most glycosylation sites were resolved chromatographically with the aforementioned overlaps efficiently resolved by the mass spectrometric dimension. IgG and IgA glycans were qualitatively in line with previous reports (supplemental Fig. S3) (20). Minor quantitative differences are consistent with the large age difference of the two sets of healthy donors. Of note, we detected more glycoforms than in the previous report, due to an increased spectral quality. We attribute this to the omission of a detergent for denaturation which likely circumvented losses from the detergent precipitation step and reduced LC-MS performance from residual detergent. Furthermore, we detect larger sialylated glycans on IgA_N47, suggesting a slightly improved proteolytic cleavage efficiency.

Overall, our IgM glycosylation data is qualitatively and quantitatively consistent with reports by Pabst *et al*, though a comparison of IgM_N209 is complicated by partially incorrect glycan assignment (15). We observed more sialylation of IgM_N209 and JC_ N71 which is easily explained by partial post-source decay of labile sialylated glycoforms in the previous MALDI-MS method which did not employ sialic acids derivatization (29). There is a noticeable difference between joining chain data from IgM (Pabst *et al* (15)), combined IgM + IgA (our study), and IgA (Momcilovic *et al* (20)) in the presence of hybrid-type glycans, reported to be absent, minor structures or major structures, respectively. This warrants further investigation, as strong differences in IgA- and IgM-derived joining chain glycosylation may be significant for function or may enable to quantitatively distinguish origins in mixtures.

### Associations with Pregnancy and Rheumatoid Arthritis

The analysis of a subset of the PARA cohort confirmed the method's suitability for the analysis of large clinical cohorts. Next to observing a low technical variation (supplemental Fig. S3), we could recapitulate findings of Ig-related glycosylation traits previously reported in the same subset (Fig. 4, *A* and *E*) (22). Our findings regarding IgG were also in line with equally subclass-resolved reports on the whole PARA cohort (30). Equally, for IgA N-glycans, our study results matched previous reports both regarding pregnancy- and RA associations (31, 32). Diverging associations with IgA1/2_N340/327 antennarity imply that pregnancy could impact C-terminal tyrosine-clipping. Negative associations of IgA O-sialylation with pregnancy and RA contradicted previous observations (31, 32). Furthermore, IgA O-galactosylation and IgA O-glycosylation site occupancy showed previously unreported, negative associations with RA. As the effect sizes are relatively small compared to the biological variation, the most likely explanation is that our method, which puts a stronger focus on resolution, allows us to see smaller effects than the method used in previous studies which emphasized throughput more strongly. However, in line with our findings, a recent integrative, post-acquisition analysis showed highly galactosylated and sialylated IgA O-glycan to be decreased in RA (33). We found various associations of IgM glycosylation with pregnancy and RA which are almost entirely novel and provide direct evidence for a strong positive association of IgM bisection with RA (22). Especially interesting are the opposing correlations of core or antennary fucosylation with both pregnancy and RA, minding method limitations. Other notable correlations can be roughly summarized as changes in the overall processing state. While previous studies went much deeper into the matter of pregnancy-induced amelioration of RA, this is not within our study's scope. In addition, due to the relatively small sample size of the study, it could not be determined whether the observed differences between RA patients and healthy controls are related to RA itself, disease activity, or the medication used to treat RA. Nonetheless, there is an interesting parallel between the larger pregnancy-associated increase in IgG1 galactosylation in RA patients versus healthy controls with the strong negative association of IgG-Fc-associated galactosylation with disease activity previously reported in the same sample set (22).

Interestingly, major galactosylation and sialylation associations are shared between isotypes. This suggests that the responsible control mechanisms affect B cells, at least to some extent, independently of class switching. Still, since the functional impact of constant domain glycosylation changes is not (fully) shared between isotypes, similar glycosylation changes may divergently affect the isotypes’ effector functions. In contrast, we observed that sometimes an isotype or even a single site showed opposing associations for a glycosylation trait. IgA, mainly its O-glycosylation, but also to a lesser extent its N-glycosylation seemed prominent in this. The differential regulation of O-glycans is not surprising, especially given the two different biosynthetic enzymes forming the, respectively, dominant α2,3- and α2,6-linkages of N-acetylneuraminic acid on O-glycans and N-glycans. Significant structural differences between the isotypes and glycosylation sites likely explain the other instances of differential associations, with the simultaneous presence of monomers and dimers further complicating the situation for IgA. Together, the differential impact on effector functions and the partially diverging regulation, require a comprehensive and isotype-resolved monitoring of antibody glycosylation.

## Conclusion

Our study presents a precise and robust method for the site-specific analysis of IgM glycosylation with unprecedented depth of glycoform coverage. Importantly, our method combines IgG, IgA, and IgM glycosylation analysis into a single measurement achieving great isotype, subclass, and site resolution in a very efficient manner. We demonstrate this by recapitulating previous findings as well as presenting novel ones, for example regarding different glycan types on IgM glycosylation sites. Application to a large clinical sample set proves the utility of our method for such purposes. Associations with pregnancy and RA, while partially previously reported, are demonstrated with superior isotype, subclass, site, and glycoform resolution and/or coverage. Especially our findings relating to N-glycan site occupancy and early N-glycan processing would not have been evident in previous approaches. The high sensitivity, optimized throughput and automation of data processing of our integrated approach allows for a fast and comprehensive analysis of IgG/A/M glycosylation from minimal amounts of precious patient material. In the future, we hope to demonstrate that the workflow is applicable to glycosylation analysis of antigen-specific IgM and even a comprehensive isotype-resolved coverage of complex antibody responses (26).

## Data Availability

The glycopeptide identification and quantitation data underlying this manuscript are publicly available on ProteomeXchange (PXD057528) via the PRIDE repository (25).

## Supporting Information

A document with Supplementary Figures – SDS-PAGE results, extracted ion chromatograms, full scan mass spectra, fragmentation spectra, precision data, and results of the cohort analysis – and Supplementary Methods – a table with chemicals and enzymes, and descriptions of LC-MS and data processing method details – (DOCX); tables with processing parameters, calculations, tryptic peptides, the raw data, and detailed statistical outcomes relating to Fig. 4, supplemental Figs. S3 and S5 (XLSX).

## Conflicts of interest

The authors declare that they have no conflicts of interest with the contents of this article.

## References

[1] Arnold J.N., Wormald M.R., Sim R.B., Rudd P.M., Dwek R.A. (2007). The impact of glycosylation on the biological function and structure of human immunoglobulins. Annu. Rev. Immunol..

[2] de Haan N., Falck D., Wuhrer M. (2020). Monitoring of immunoglobulin N- and O-glycosylation in health and disease. Glycobiology.

[3] Bondt A., Hafkenscheid L., Falck D., Kuijper T.M., Rombouts Y., Hazes J.M.W. (2018). ACPA IgG galactosylation associates with disease activity in pregnant patients with rheumatoid arthritis. Ann. Rheum. Dis..

[4] Larsen M.D., de Graaf E.L., Sonneveld M.E., Plomp H.R., Nouta J., Hoepel W. (2021). Afucosylated IgG characterizes enveloped viral responses and correlates with COVID-19 severity. Science.

[5] Gudelj I., Lauc G., Pezer M. (2018). Immunoglobulin G glycosylation in aging and diseases. Cell Immunol..

[6] Ackerman M.E., Crispin M., Yu X., Baruah K., Boesch A.W., Harvey D.J. (2013). Natural variation in Fc glycosylation of HIV-specific antibodies impacts antiviral activity. J. Clin. Invest..

[7] Lai K.N., Tang S.C.W., Schena F.P., Novak J., Tomino Y., Fogo A.B. (2016). IgA nephropathy. Nat. Rev. Dis. Primers.

[8] Hiki Y., Odani H., Takahashi M., Yasuda Y., Nishimoto A., Iwase H. (2001). Mass spectrometry proves under-O-glycosylation of glomerular IgA1 in IgA nephropathy. Kidney Int..

[9] Steffen U., Koeleman C.A., Sokolova M.V., Bang H., Kleyer A., Rech J. (2020). IgA subclasses have different effector functions associated with distinct glycosylation profiles. Nat. Commun..

[10] Basset C., Durand V., Jamin C., Clément J., Pennec Y., Youinou P. (2000). Increased N-linked glycosylation leading to oversialylation of monomeric immunoglobulin A1 from patients with Sjogren's syndrome. Scand. J. Immunol..

[11] Beyer H., Sommerfeld M., Grandien K., Faust C., Tillmann B., Leuschner W.D. (2024). Functional studies with IgM and IgA immunoglobulins: binding to pIgR, FcalphamuR, FcmuR, and CDC activities. APMIS.

[12] Dati F., Schumann G., Thomas L., Aguzzi F., Baudner S., Bienvenu J. (1996). Consensus of a group of professional societies and diagnostic companies on guidelines for interim reference ranges for 14 proteins in serum based on the standardization against the IFCC/BCR/CAP Reference Material (CRM 470). International Federation of Clinical Chemistry. Community Bureau of Reference of the Commission of the European Communities. College of American Pathologists. Eur. J. Clin. Chem. Clin. Biochem..

[13] William R.S., Strohl L.M., William R.S., Strohl L.M. (2012). Therapeutic Antibody Engineering.

[14] Hong Q., Ruhaak L.R., Stroble C., Parker E., Huang J., Maverakis E. (2015). A method for comprehensive glycosite-mapping and direct quantitation of serum glycoproteins. J. Proteome Res..

[15] Pabst M., Küster S.K., Wahl F., Krismer J., Dittrich P.S., Zenobi R. (2015). A microarray-matrix-assisted laser desorption/ionization-mass spectrometry approach for site-specific protein N-glycosylation analysis, as demonstrated for human serum immunoglobulin M (IgM). Mol. Cell Proteomics.

[16] Arnold J.N., Wormald M.R., Suter D.M., Radcliffe C.M., Harvey D.J., Dwek R.A. (2005). Human serum IgM glycosylation: identification of glycoforms that can bind to mannan-binding lectin. J. Biol. Chem..

[17] Chandler K.B., Mehta N., Leon D.R., Suscovich T.J., Alter G., Costello C.E. (2019). Multi-isotype glycoproteomic characterization of serum antibody heavy chains reveals isotype- and subclass-specific N-glycosylation profiles. Mol. Cell Proteomics.

[18] Moh E.S., Lin C.H., Thaysen-Andersen M., Packer N.H. (2016). Site-specific N-glycosylation of recombinant pentameric and hexameric human IgM. J. Am. Soc. Mass Spectrom..

[19] Cheng Y.H., Lee C.H., Wang S.Y., Chou C.Y., Yang Y.J., Kao C.C. (2024). Multiplexed antibody glycosylation profiling using dual enzyme digestion and liquid chromatography-triple quadrupole mass spectrometry method. Mol. Cell Proteomics.

[20] Momcilovic A., de Haan N., Hipgrave Ederveen A.L., Bondt A., Koeleman C.A.M., Falck D. (2020). Simultaneous immunoglobulin A and G glycopeptide profiling for high-throughput applications. Anal. Chem..

[21] de Man Y.A., Dolhain R.J.E.M., van de Geijn F.E., Willemsen S.P., Hazes J.M.W. (2008). Disease activity of rheumatoid arthritis during pregnancy: results from a nationwide prospective study. Arthritis Rheum..

[22] Reiding K.R., Bondt A., Hennig R., Gardner R.A., O'Flaherty R., Trbojević-Akmačić I. (2019). High-throughput serum N-glycomics: method comparison and application to study rheumatoid arthritis and pregnancy-associated changes. Mol. Cell Proteomics.

[23] Amez Martin M., Wuhrer M., Falck D. (2021). Serum and plasma immunoglobulin G Fc N-glycosylation is stable during storage. J. Proteome Res..

[24] de Haan N., Pučić-Baković M., Novokmet M., Falck D., Lageveen-Kammeijer G., Razdorov G. (2022). Developments and perspectives in high-throughput protein glycomics: enabling the analysis of thousands of samples. Glycobiology.

[25] Perez-Riverol Y., Bai J., Bandla C., García-Seisdedos D., Hewapathirana S., Kamatchinathan S. (2022). The PRIDE database resources in 2022: a hub for mass spectrometry-based proteomics evidences. Nucleic Acids Res..

[26] Falck D., Wuhrer M. (2024). GlYcoLISA: antigen-specific and subclass-specific IgG Fc glycosylation analysis based on an immunosorbent assay with an LC-MS readout. Nat. Protoc..

[27] Jansen B.C., Falck D., de Haan N., Hipgrave Ederveen A.L., Razdorov G., Lauc G. (2016). LaCyTools: a targeted liquid chromatography-mass spectrometry data processing package for relative quantitation of glycopeptides. J. Proteome Res..

[28] Wojcik I., Sénard T., de Graaf E.L., Janssen G.M.C., de Ru A.H., Mohammed Y. (2020). Site-specific glycosylation mapping of Fc gamma receptor IIIb from neutrophils of individual healthy donors. Anal. Chem..

[29] de Haan N., Reiding K.R., Haberger M., Reusch D., Falck D., Wuhrer M. (2015). Linkage-specific sialic acid derivatization for MALDI-TOF-MS profiling of IgG glycopeptides. Anal. Chem..

[30] Bondt A., Selman M.H.J., Deelder A.M., Hazes J.M.W., Willemsen S.P., Wuhrer M. (2013). Association between galactosylation of immunoglobulin G and improvement of rheumatoid arthritis during pregnancy is independent of sialylation. J. Proteome Res..

[31] Bondt A., Nicolardi S., Jansen B.C., Stavenhagen K., Blank D., Kammeijer G.S.M. (2016). Longitudinal monitoring of immunoglobulin A glycosylation during pregnancy by simultaneous MALDI-FTICR-MS analysis of N- and O-glycopeptides. Sci. Rep..

[32] Bondt A., Nicolardi S., Jansen B.C., Kuijper T.M., Hazes J.M.W., van der Burgt Y.E.M. (2017). IgA N- and O-glycosylation profiling reveals no association with the pregnancy-related improvement in rheumatoid arthritis. Arthritis Res. Ther..

[33] Mayboroda O.A., Lageveen-Kammeijer G.S.M., Wuhrer M., Dolhain R.J.E.M. (2023). An integrated glycosylation signature of rheumatoid arthritis. Biomolecules.

