# Supplementary material for: Comprehensive Immunoglobulin G, A, and M Glycopeptide Profiling for Large-Scale Biomedical Research
Source: Mol Cell Proteomics. 2025 Feb 19;24(3):100928. doi: 10.1016/j.mcpro.2025.100928 (PMC11953977; doi:10.1016/j.mcpro.2025.100928)
Supplement: Supporting_Information 1 [file mmc1.docx]

Comprehensive Immunoglobulin G, A, and M glycopeptide profiling for large-scale biomedical research

**Authors:**
Bianca D. M. van Tol^1^, Anna M. Wasynczuk^1^, Steinar Gijze^1^, Oleg A. Mayboroda^1^, Jan Nouta^1^, Radboud J. E. M. Dolhain^2^, Manfred Wuhrer^1^ and David Falck^1^

^1^Center for Proteomics and Metabolomics, Leiden University Medical Center, Leiden, the Netherlands.
^2^Department of Rheumatology, Erasmus University Medical Center, Rotterdam, The Netherlands.

## Table of contents

Supplementary Figures

Figure S1: Gel of affinity purification. Page 2

Figure S2: NanoLC-qTOF-MS glycopeptide profiling of IgM. Page 3

Figure S3: Variability in the IgG/A/M glycosylation data in the PARA cohort. Page 4

Figure S4: Annotated CID fragmentation spectra of abundant IgM glycopeptides. Page 5 to 15

Figure S5: Glycosylation associations with pregnancy and rheumatoid arthritis. Page 16 to 30

Supplementary methods

Chemicals and Enzymes Page 31

Glycopeptide analysis by liquid chromatography - mass spectrometry Page 32

GlycoDash processing Page 32

## Supplementary Figures


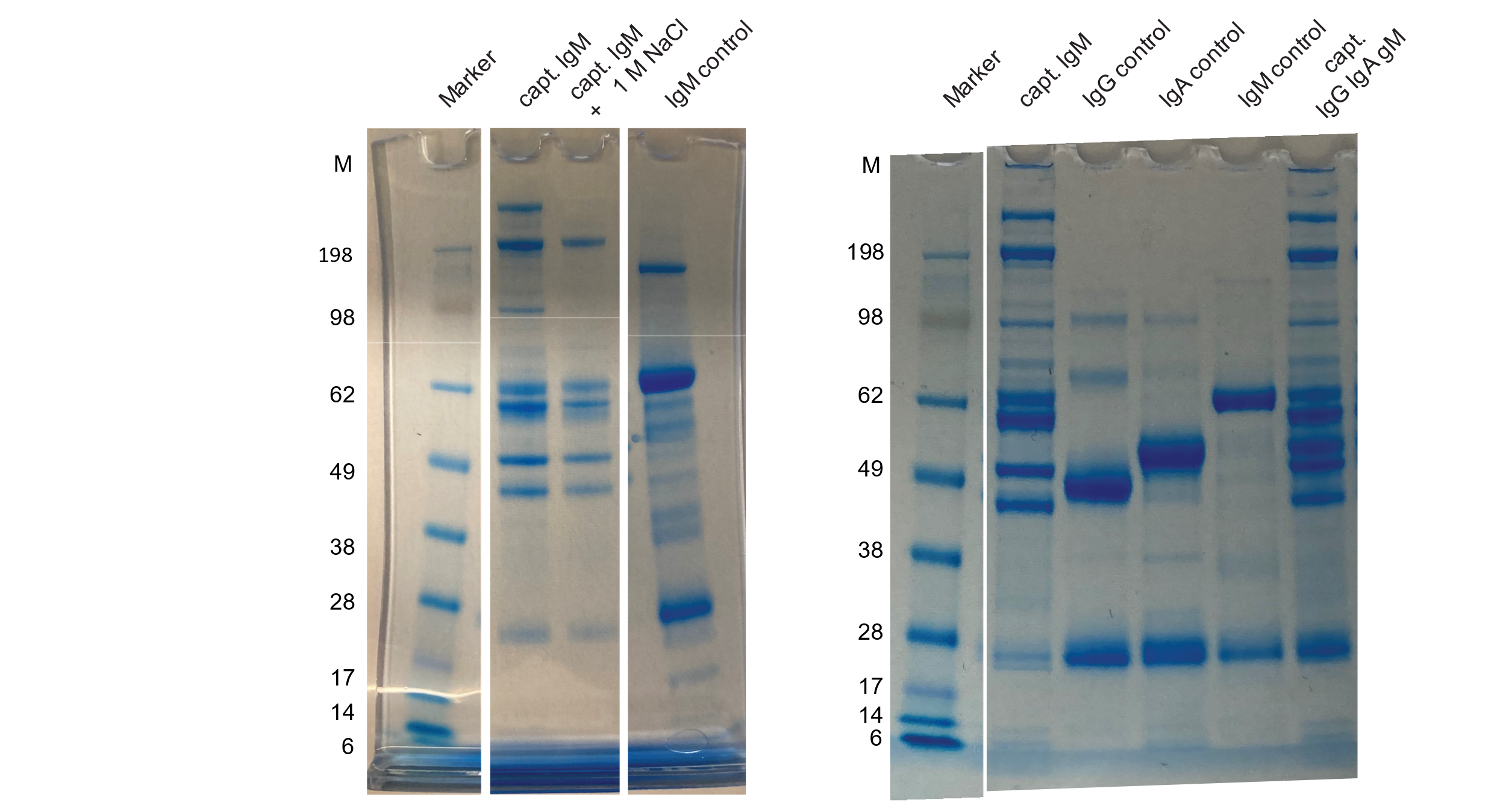


**Figure S1**: Gel of affinity purification. Differences in with and without sodium chloride in the PBS wash step.

**
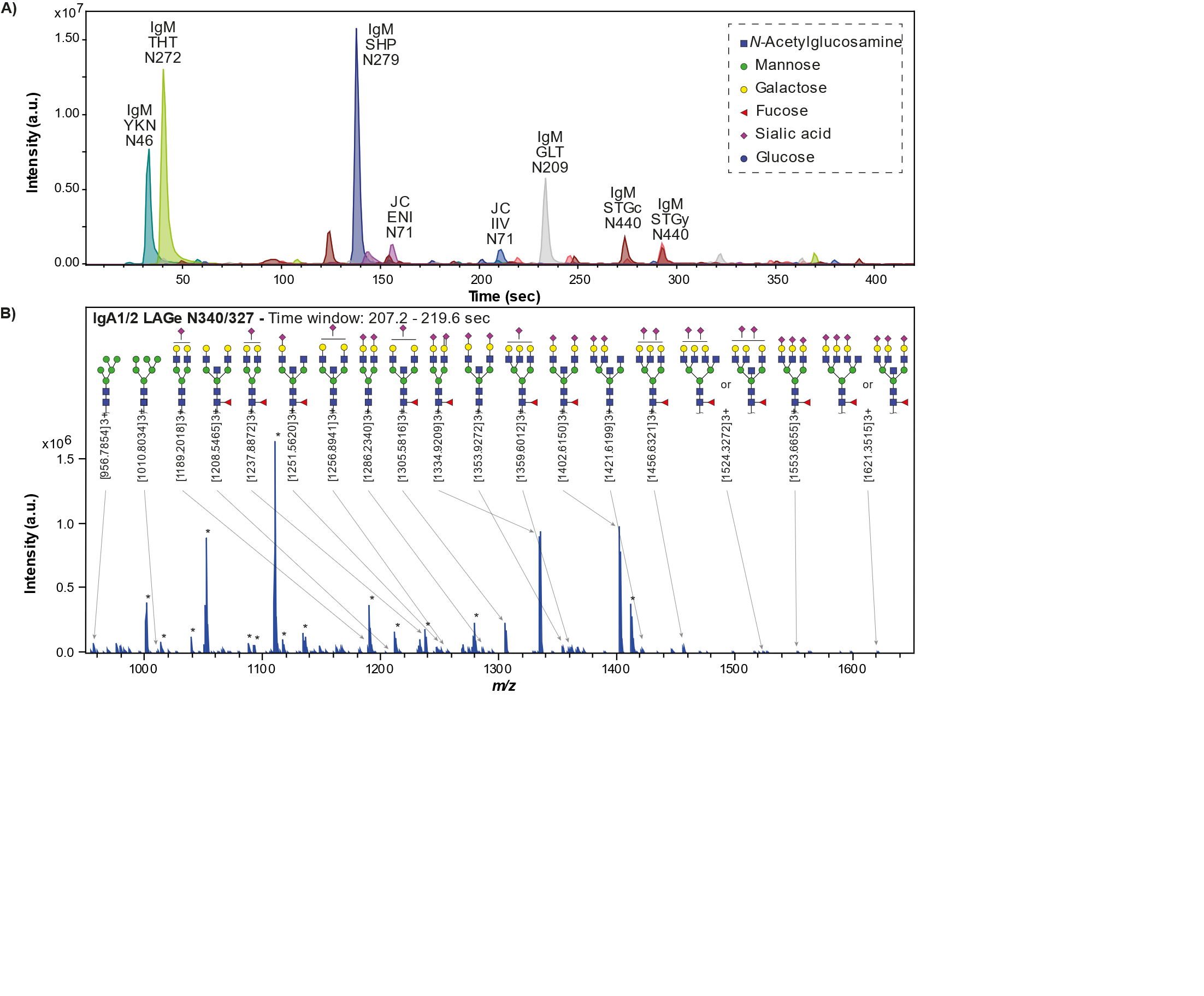
**

**Figure S2**: NanoLC-qTOF-MS glycopeptide profiling of IgM. (A) Extracted ion chromatograms (EICs) for the major glycopeptide per glycosylation site of IgM. Glycopeptides with the same peptide sequence, but different glycan structures, cluster together. (B) Annotation of the C-terminal IgA glycosylation site N340/N327 in the presence of the co-eluting joining chain glycopeptides.


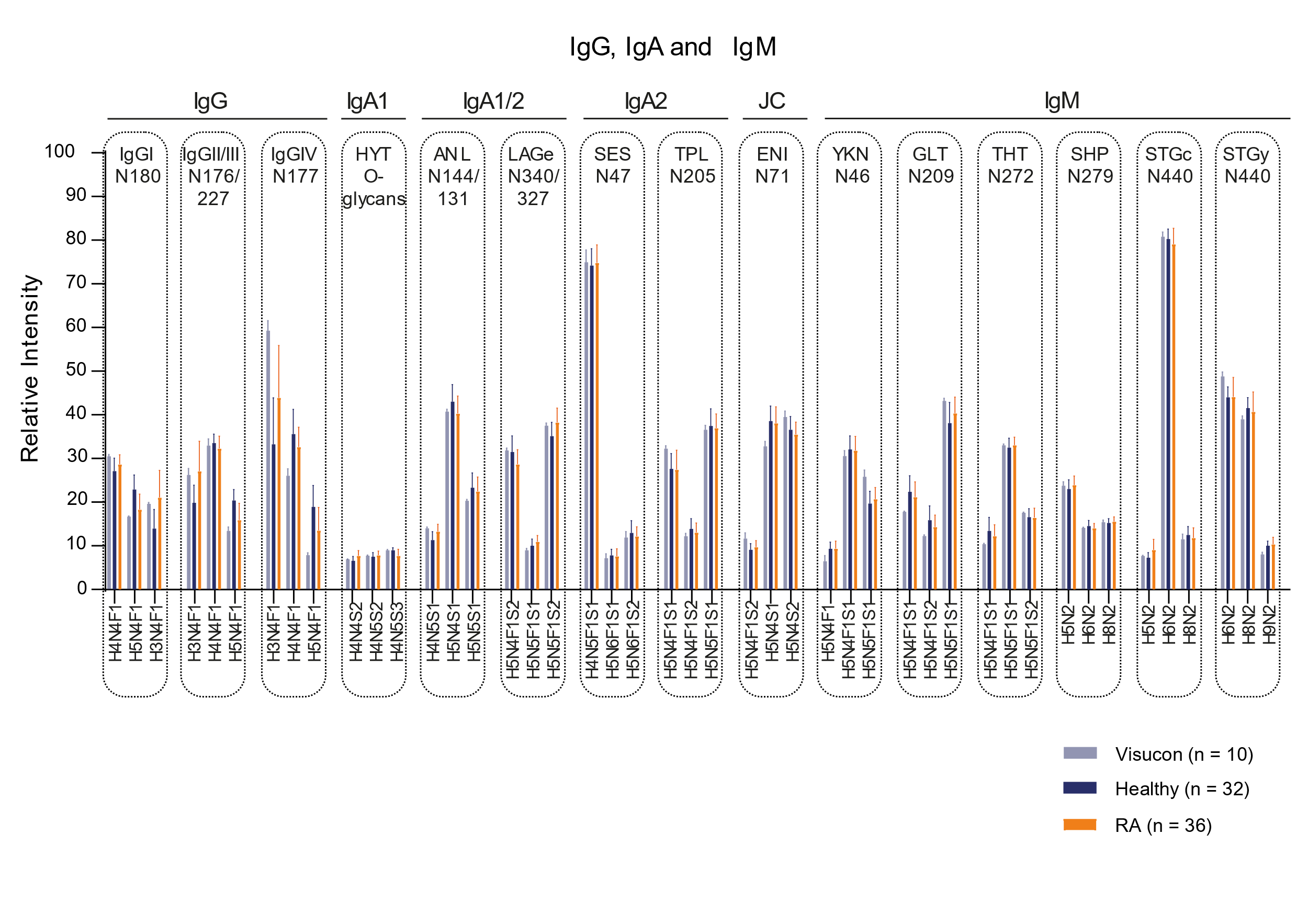


**Figure S3**: Variability in the IgG/A/M glycosylation data in the PARA cohort. Technical variability is deducible from a plasma standard (n=10). The variability in the controls (n=32) and patients (n = 36) indicate the biological variability in the healthy and disease context, respectively. Mean and standard deviation are depicted.

**A IgM N46 H5N5F1S1**

**
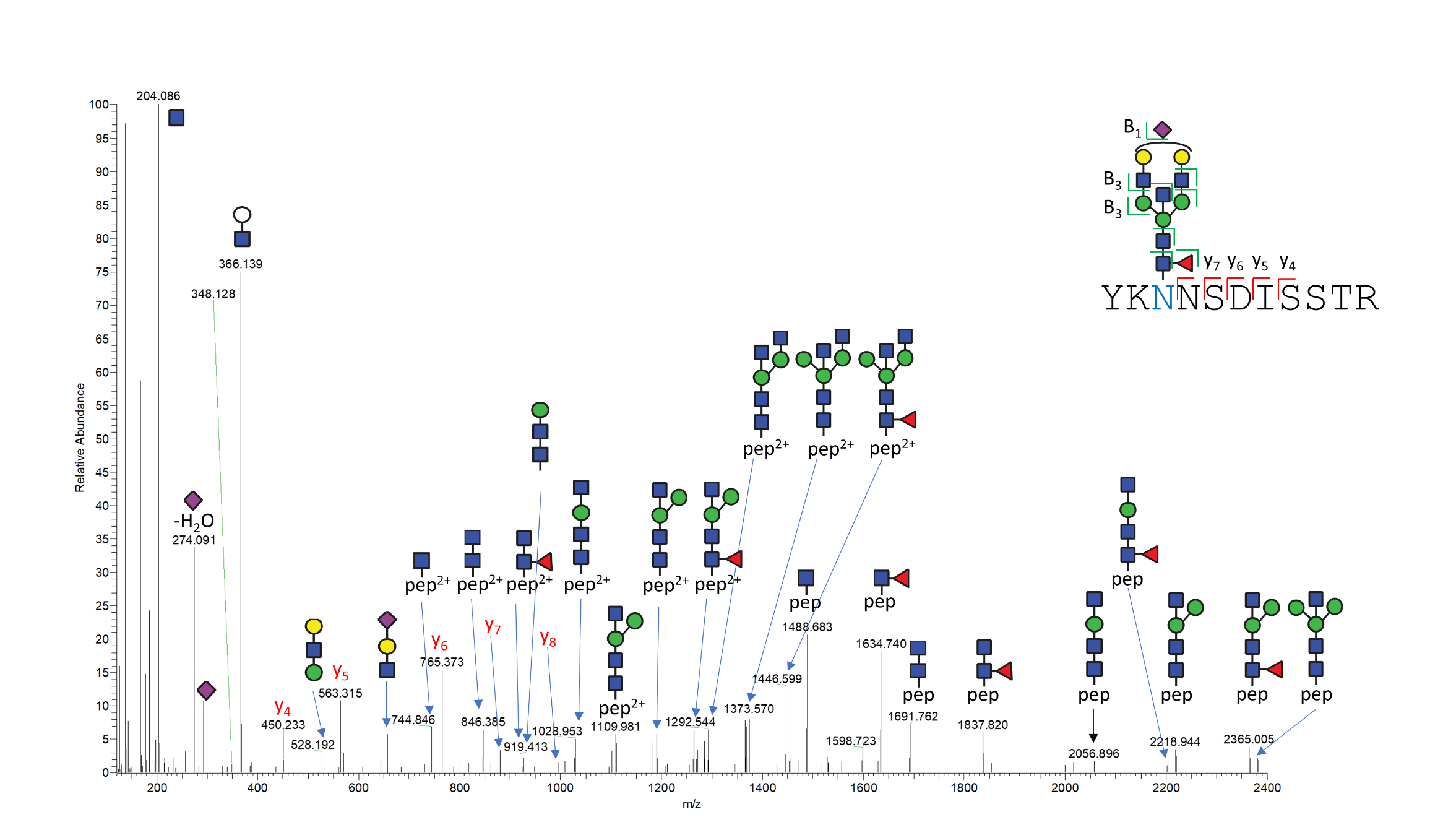
**

**B IgM N46 H5N4F1S1**

**
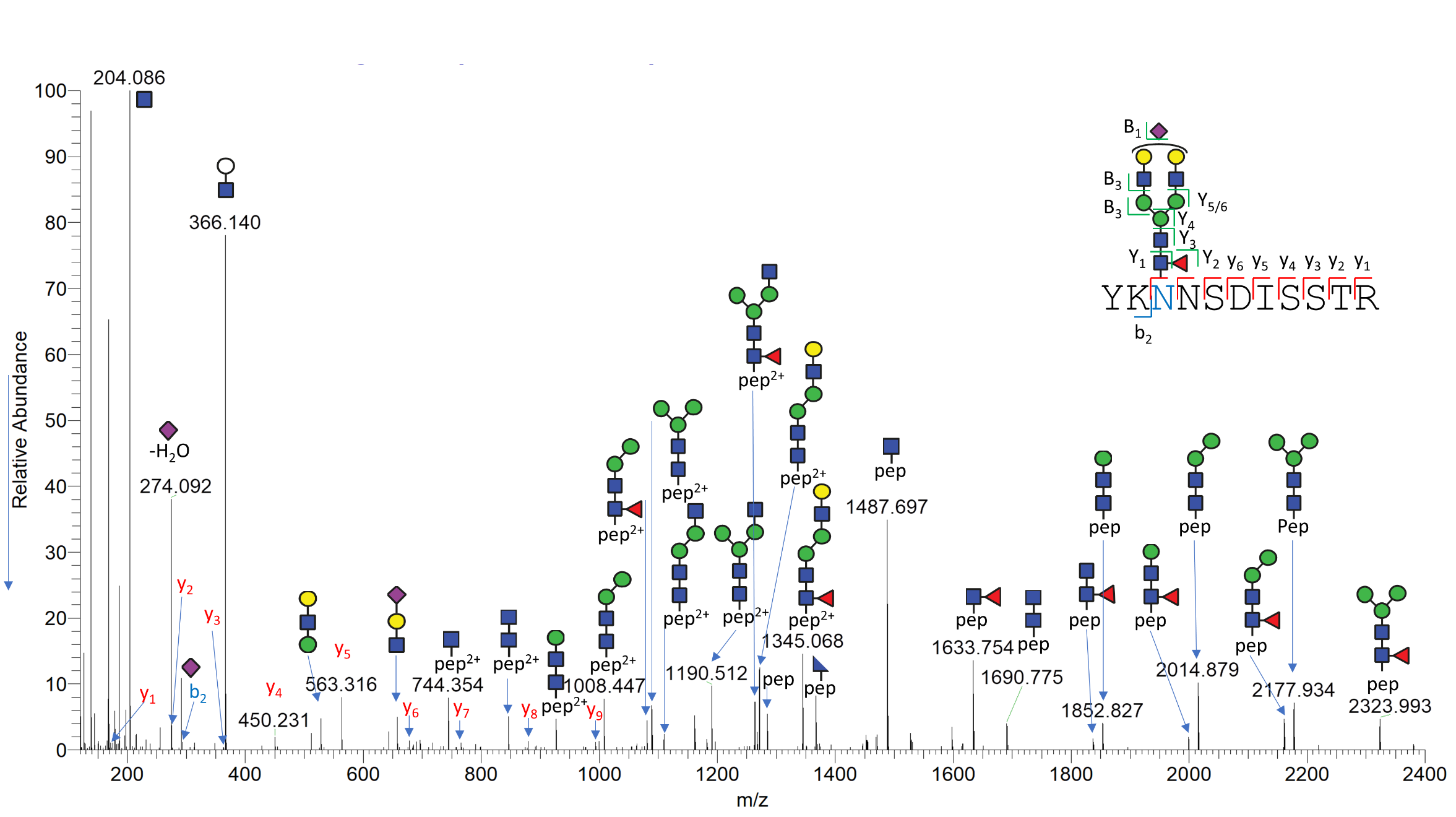
**

**C IgM N209 H5N5F1S1**

**
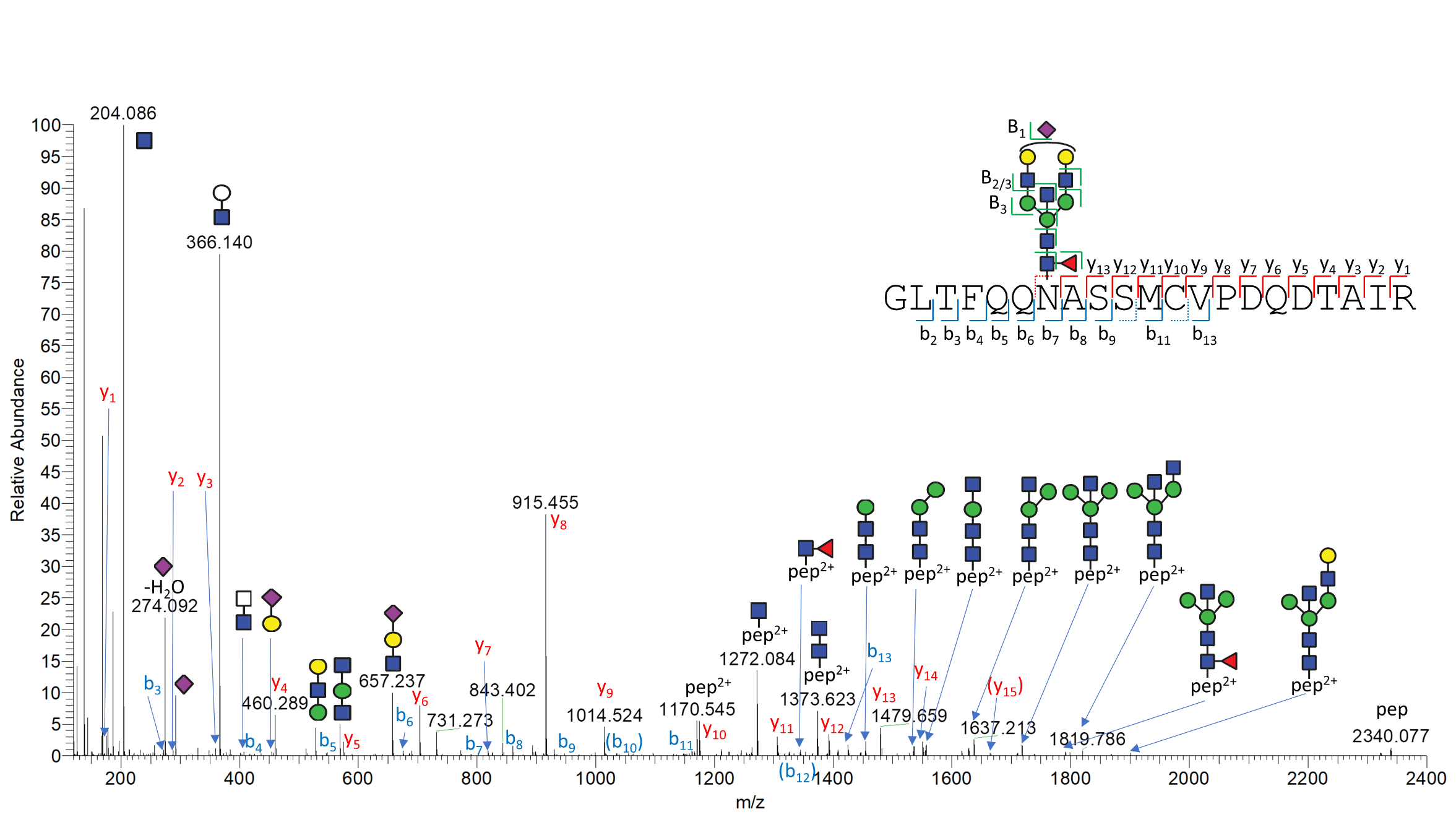
**

**D IgM N209 H5N4F1S1**


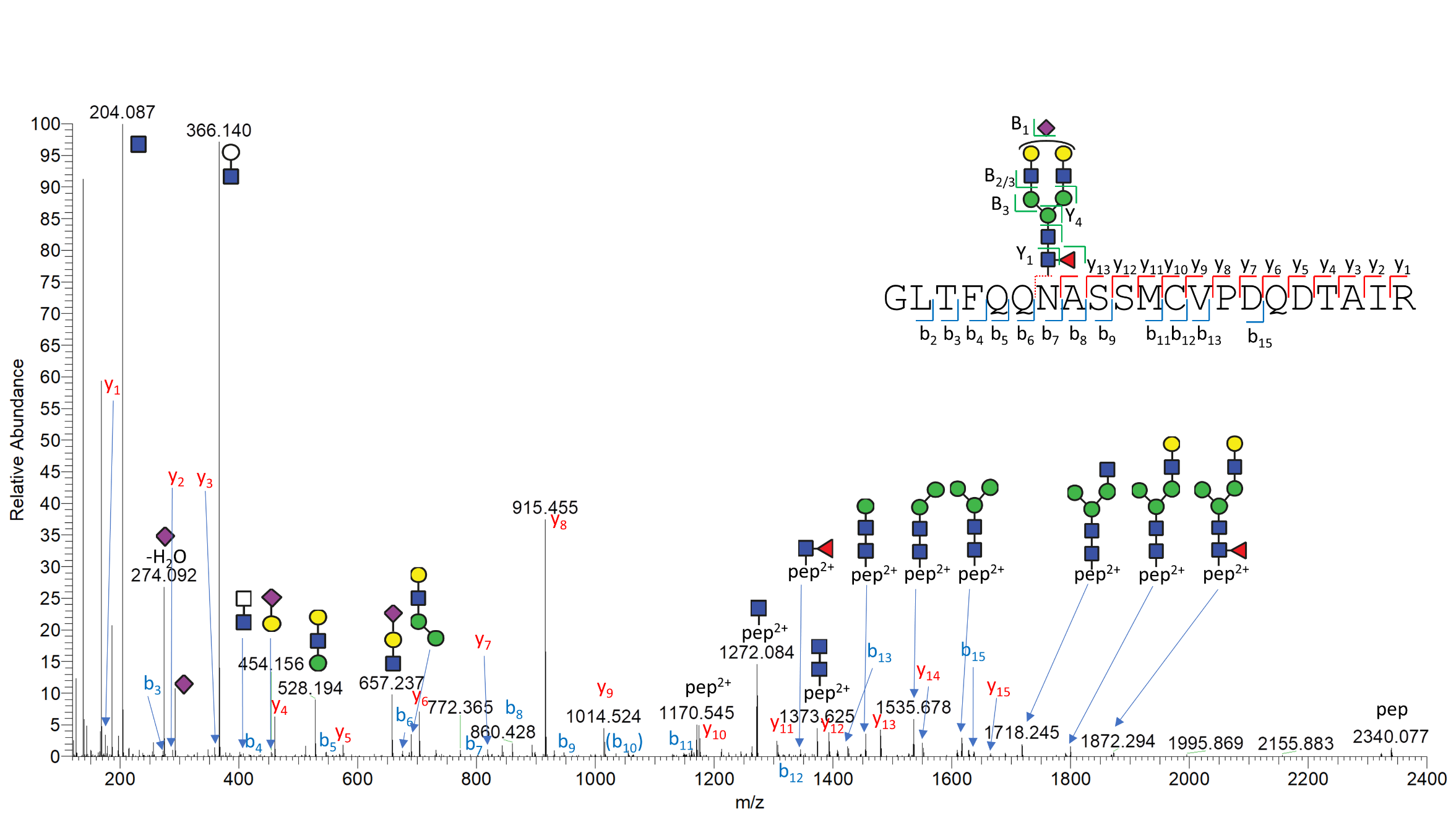


**E IgM N272 H5N5F1S1**


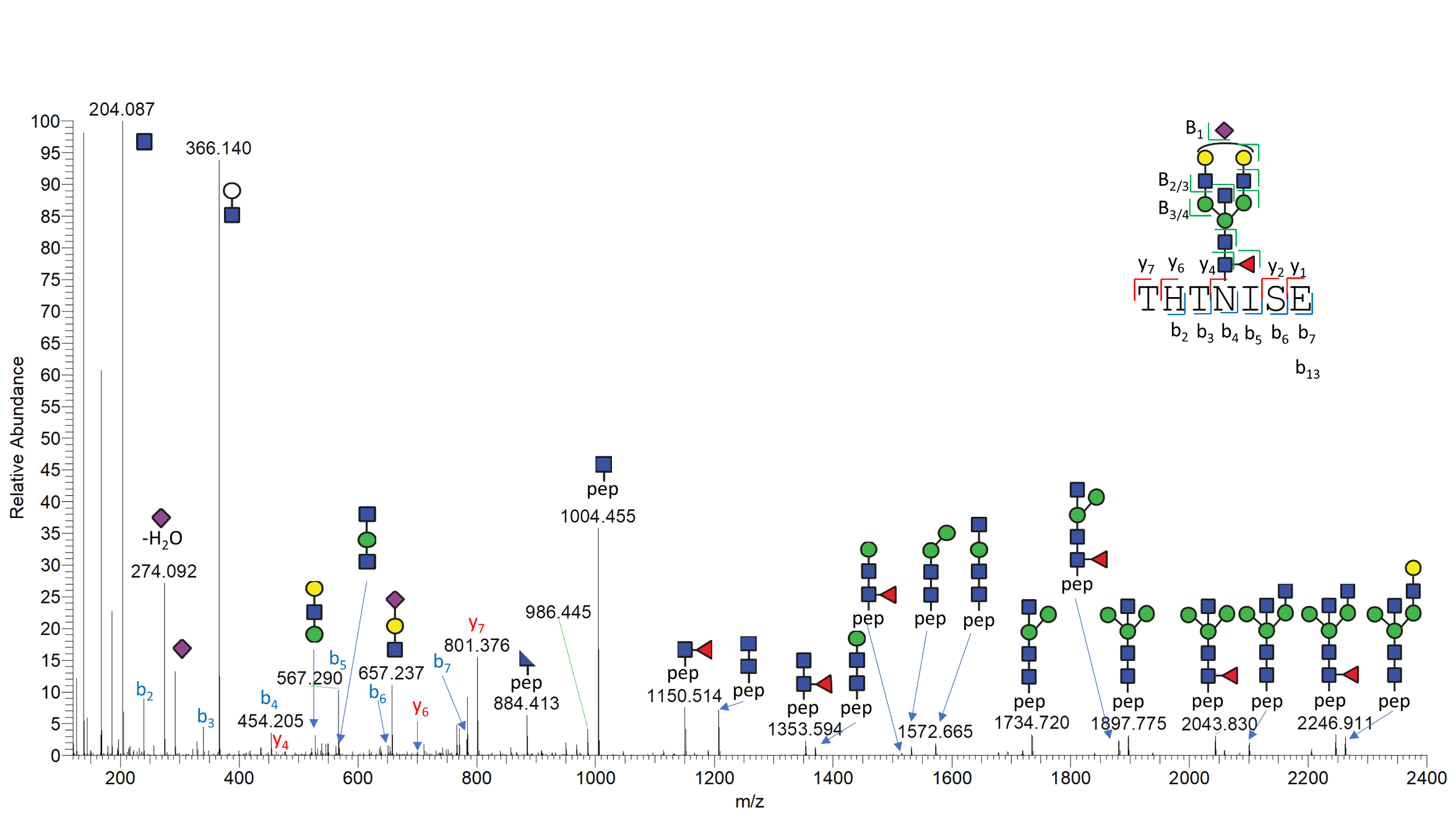


**F IgM N272 H5N5F1S2**


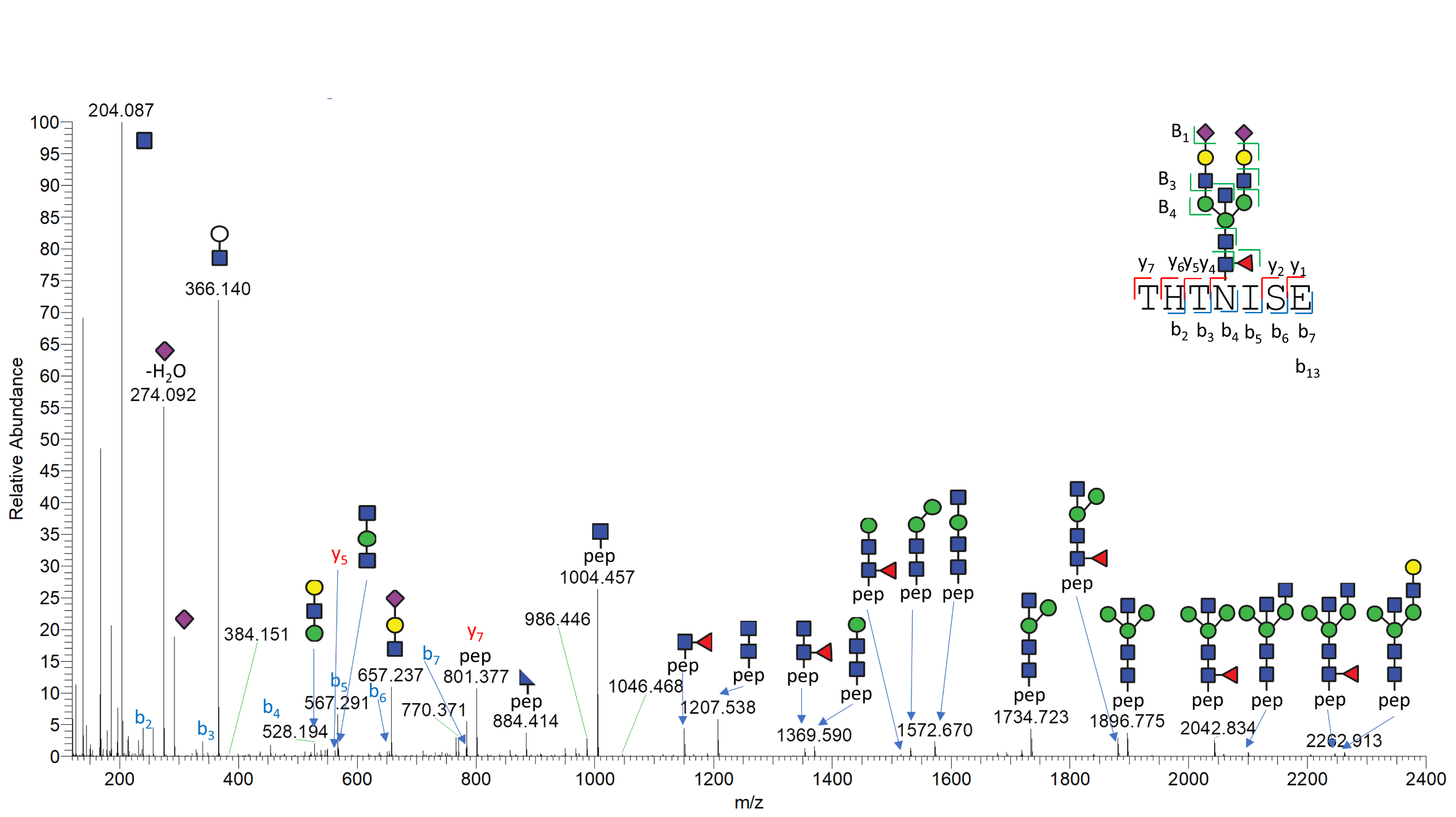


**G IgM N279 H5N2**


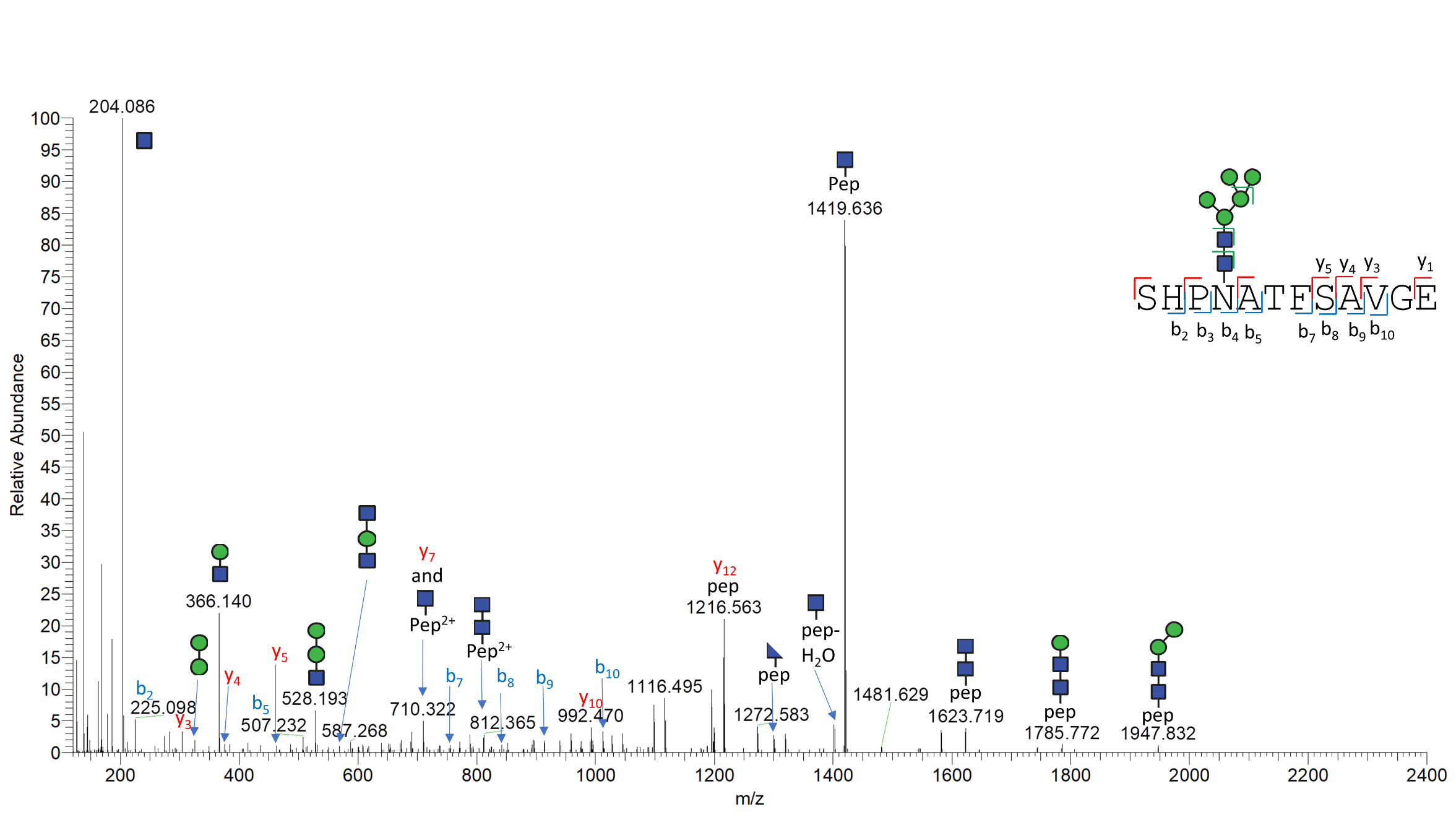


**H IgM N279 H8N2**


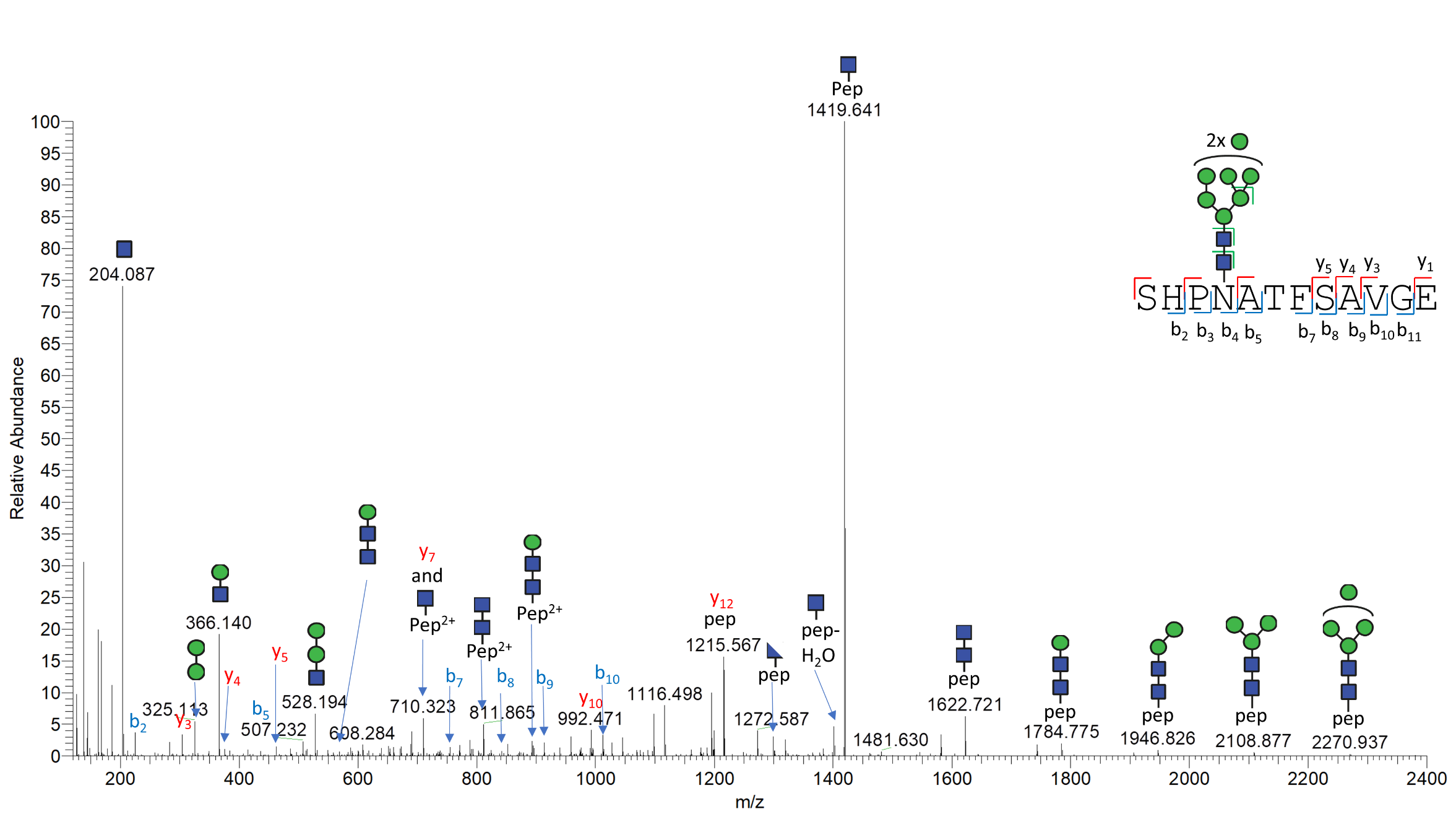


**I** **IgM N440 H6N2 (with C-terminal tyrosine cleavage)**


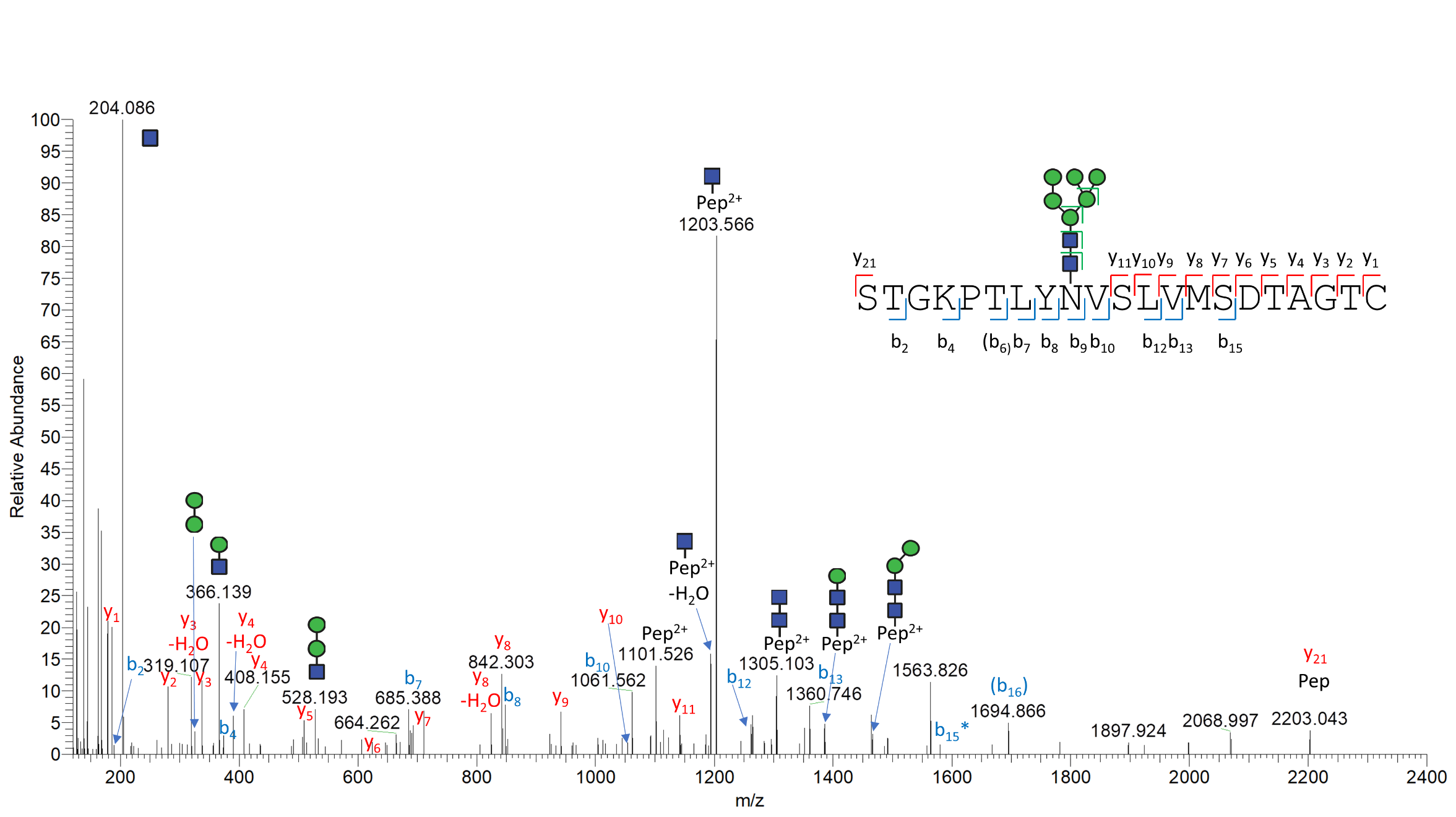


**J IgM N440 non-glycosylated (with C-terminal tyrosine cleavage)**


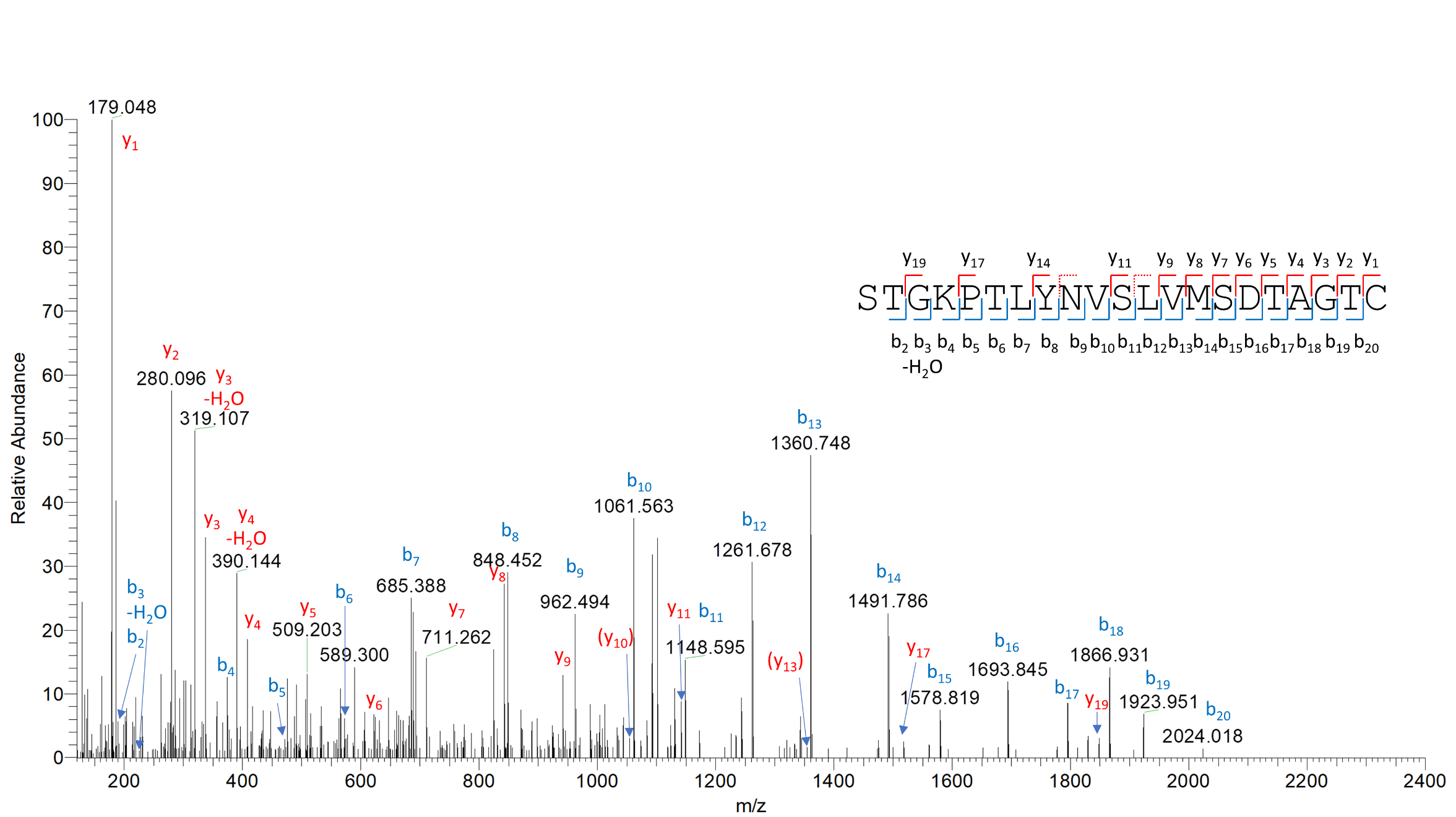


**K IgM N440 non-glycosylated (with intact C-terminal tyrosine)**


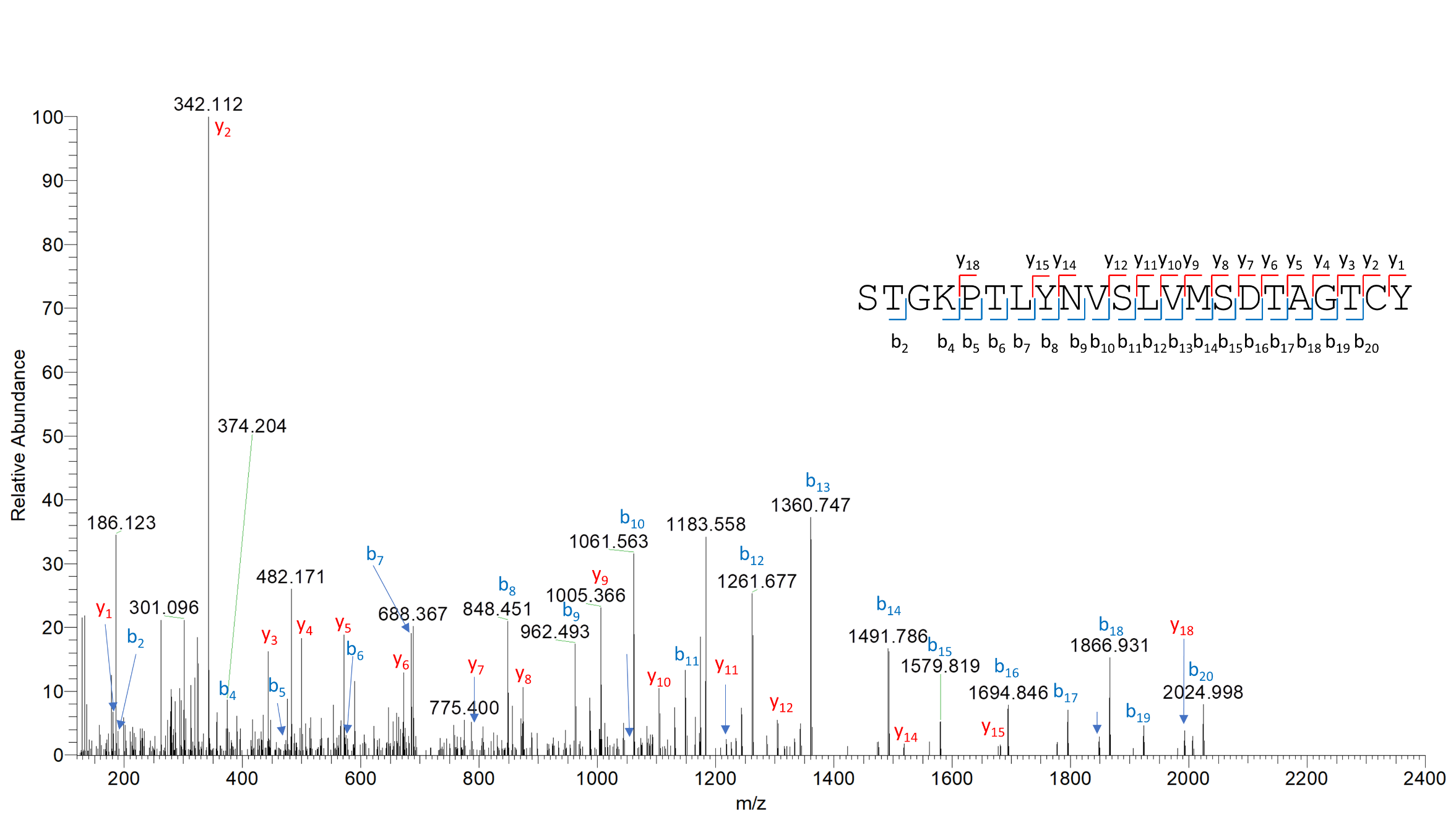


**Figure S4:** Annotated CID fragmentation spectra of abundant IgM glycopeptides. A) IgM N46 H5N5F1S1, B) IgM N46 H5N4F1S1, C) IgM N209 H5N5F1S1, D) IgM N209 H5N4F1S1, E) IgM N272 H5N5F1S1, F) IgM N272 H5N5F1S2, G) IgM N279 H5N2, H) IgM N279 H8N2, I) IgM N440 H6N2 (STGc), J) IgM N440 non-glycosylated (STGc), and K) IgM N440 non-glycosylated (STGy)


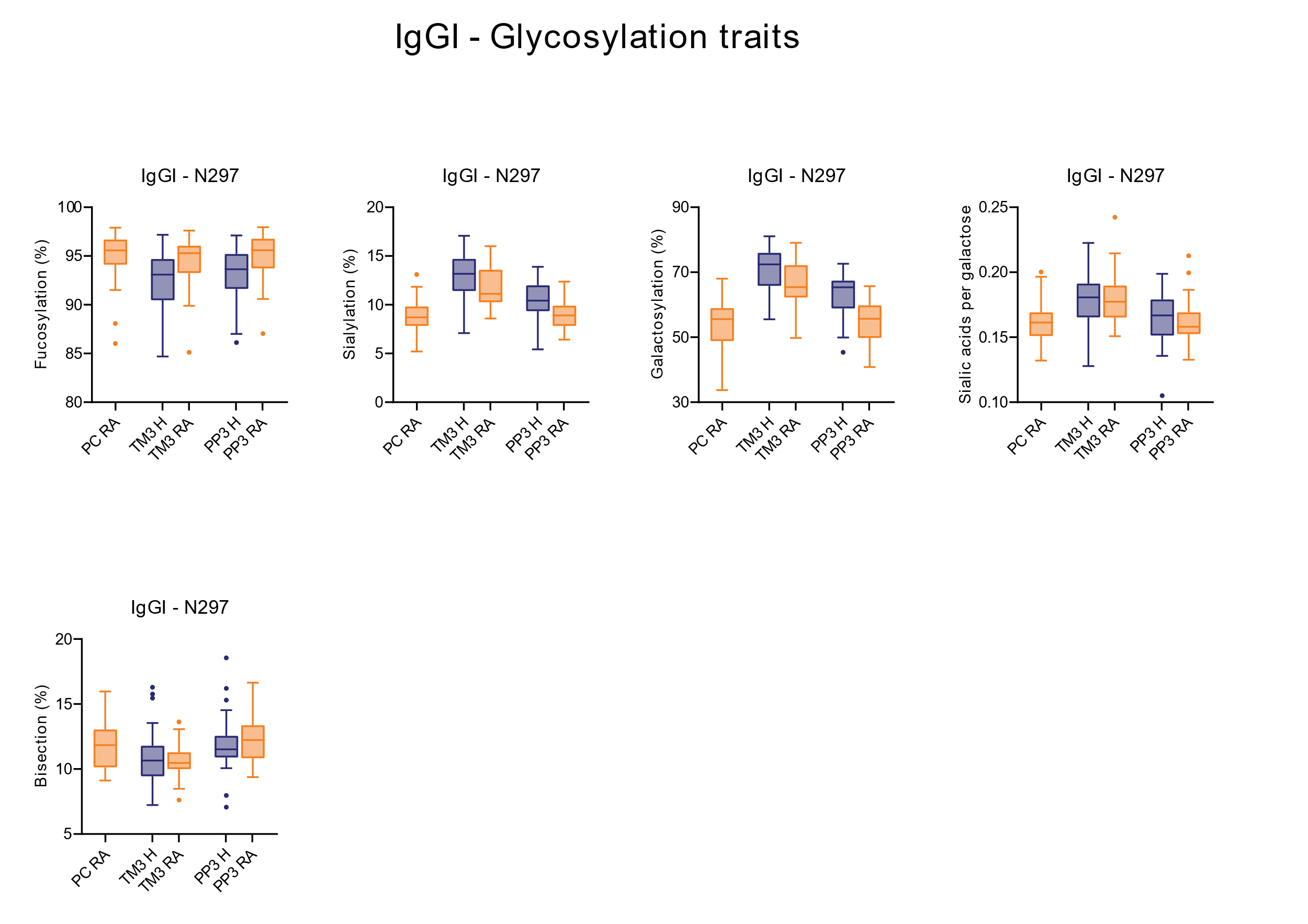

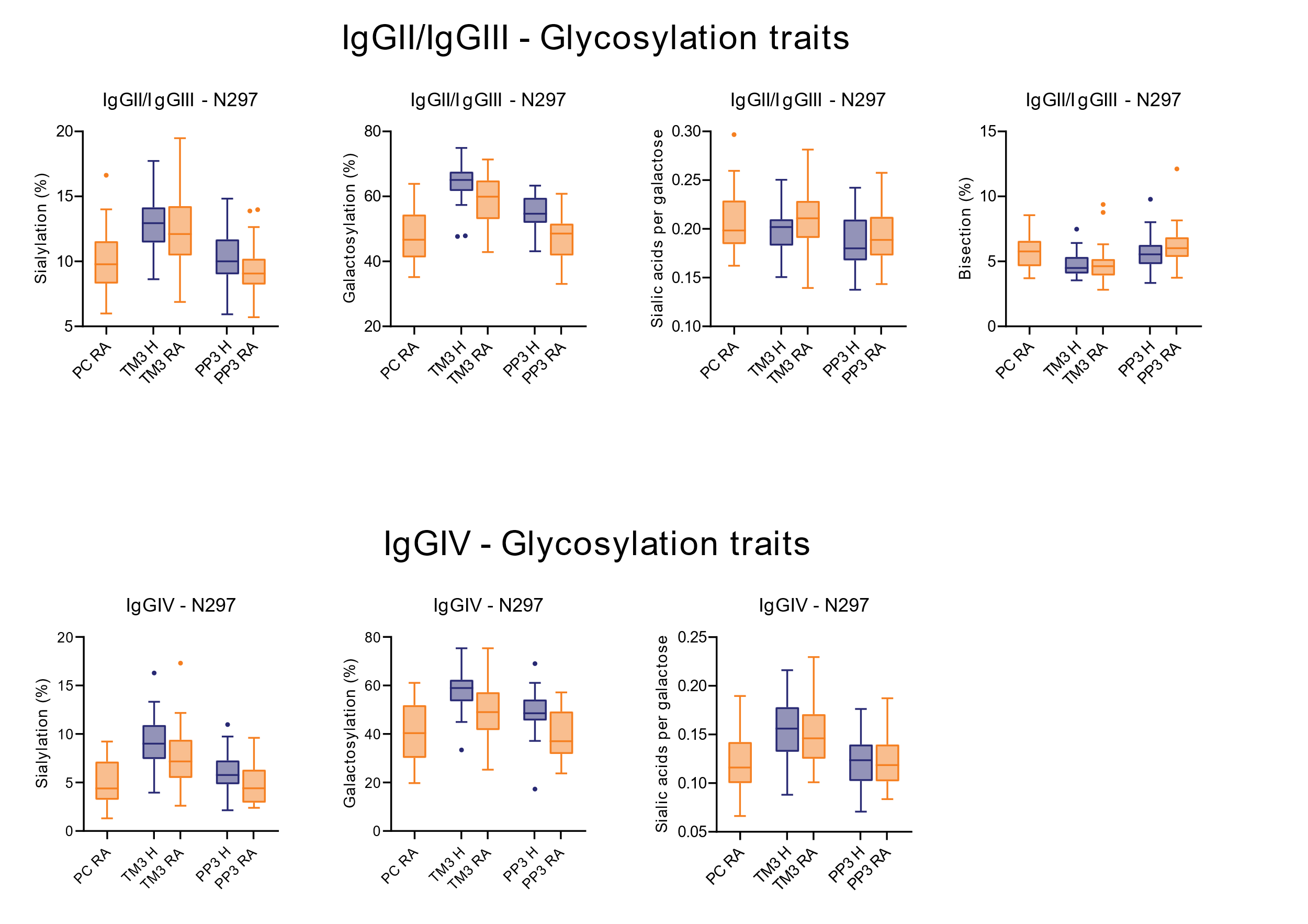

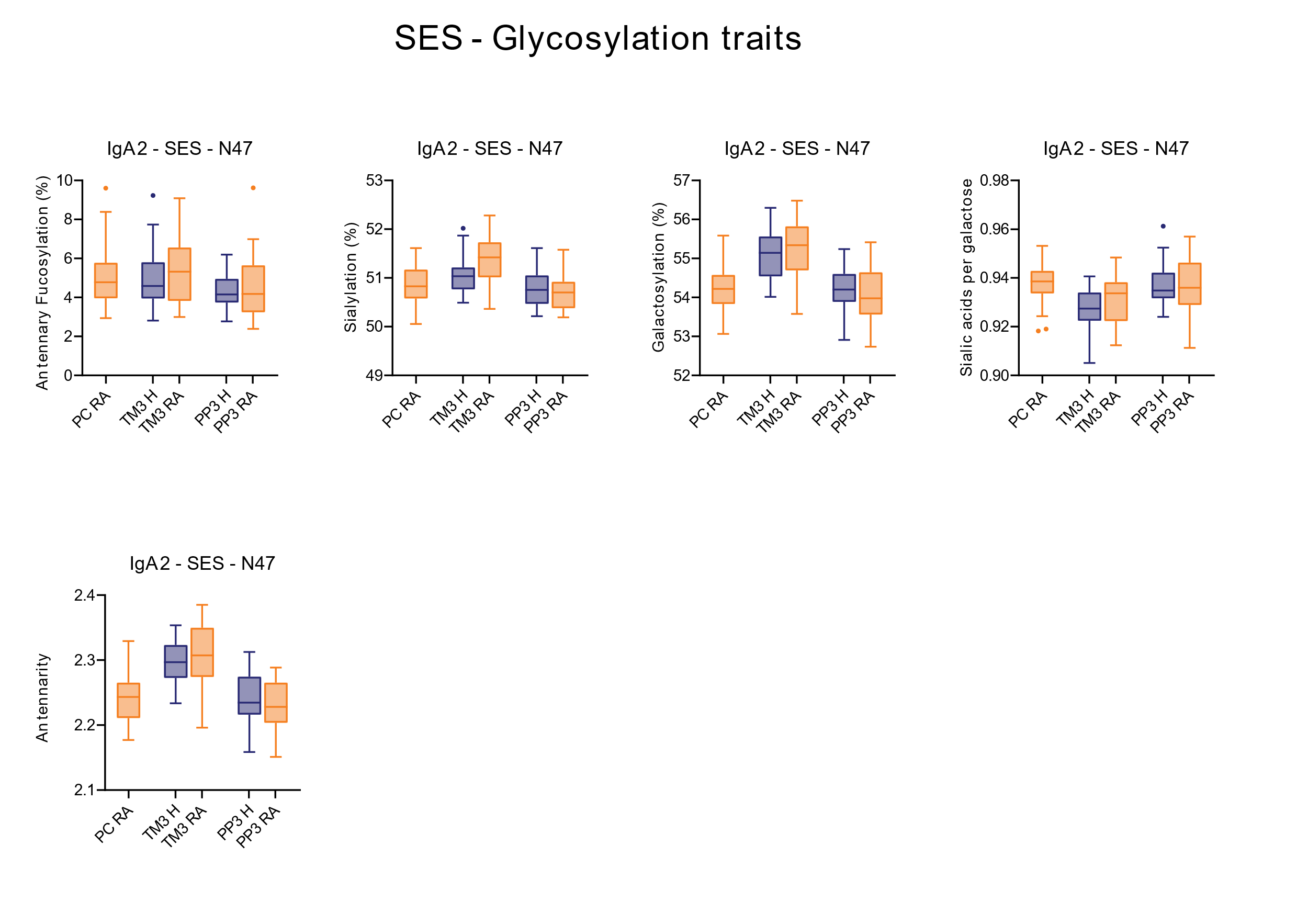

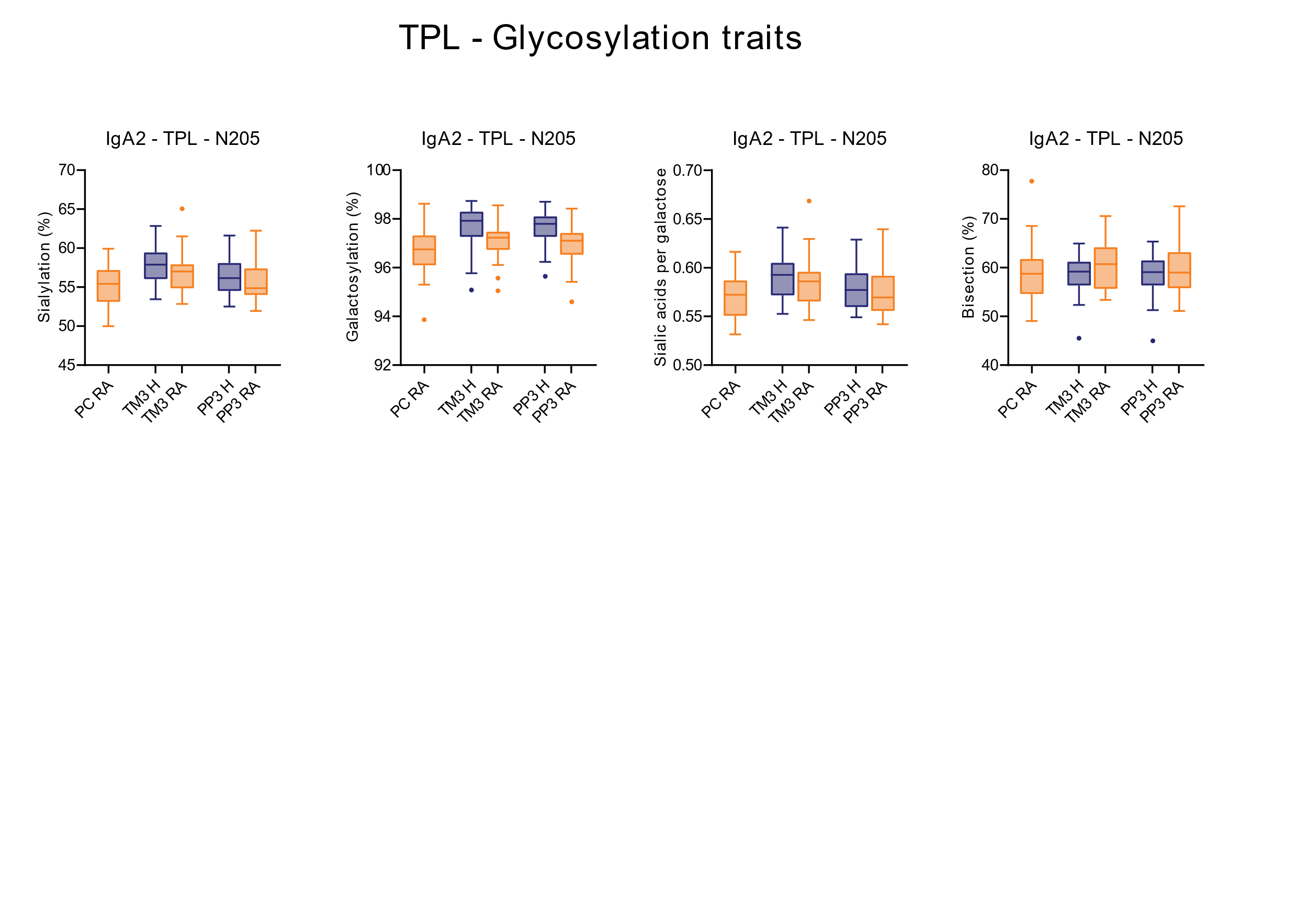

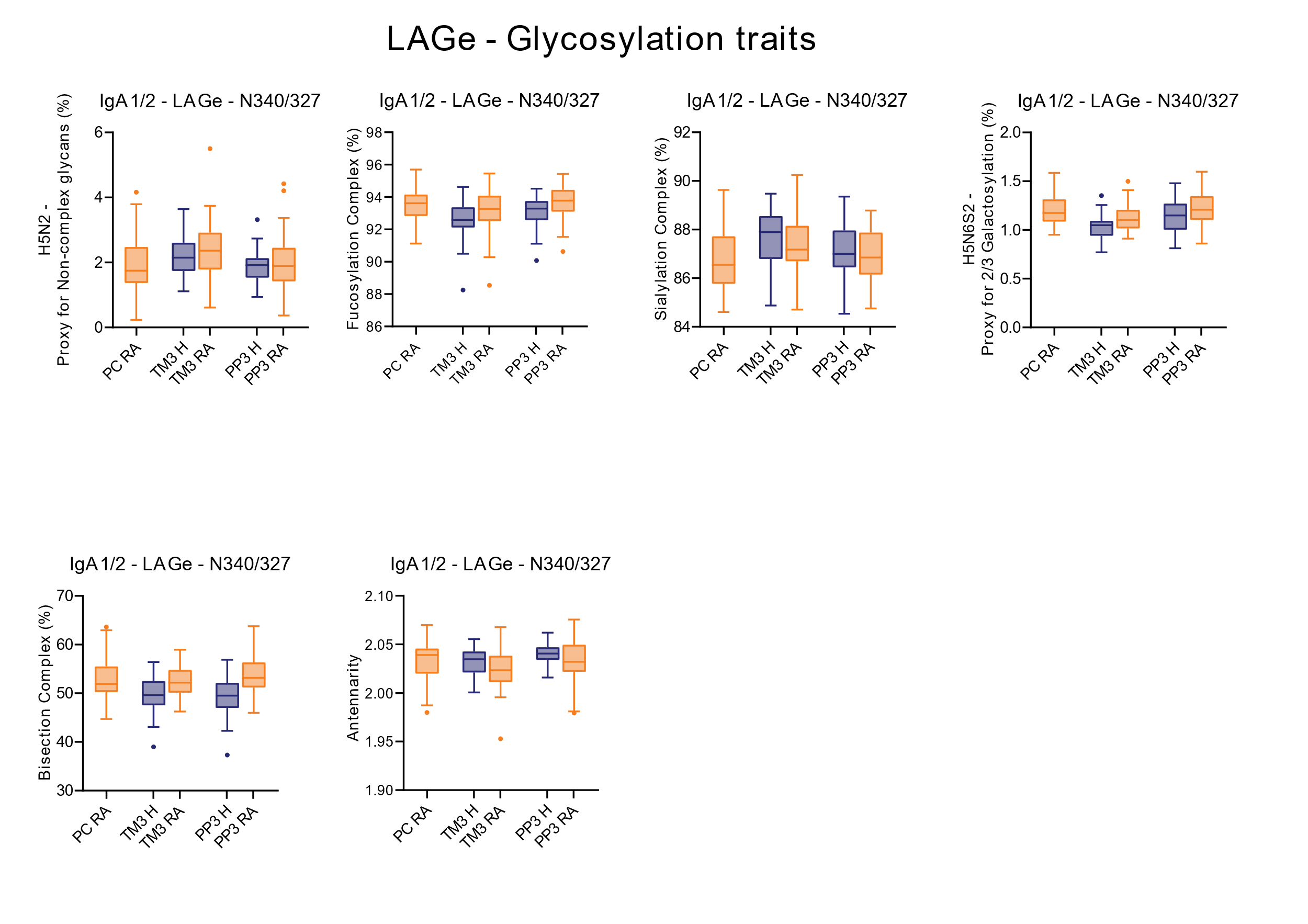

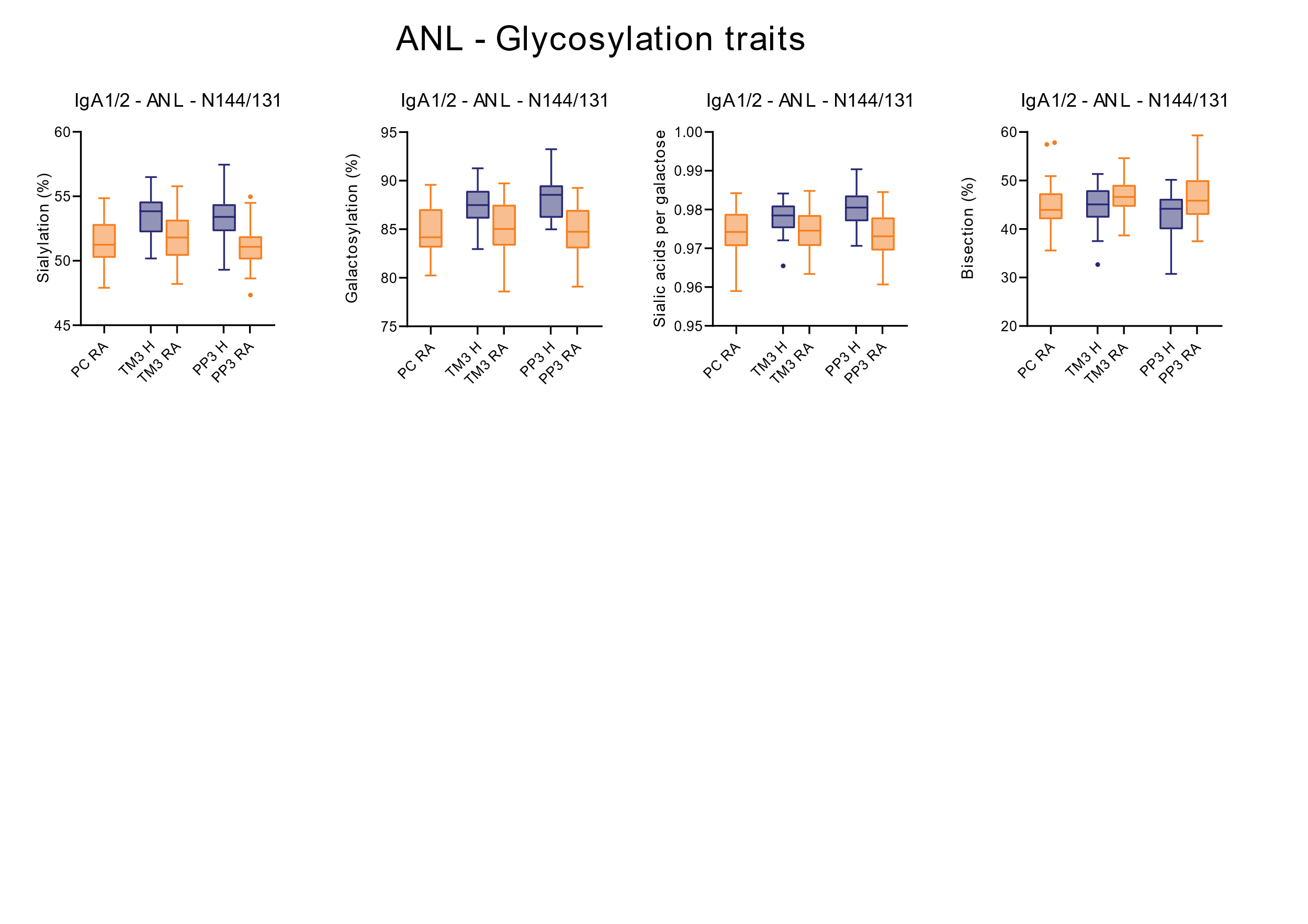

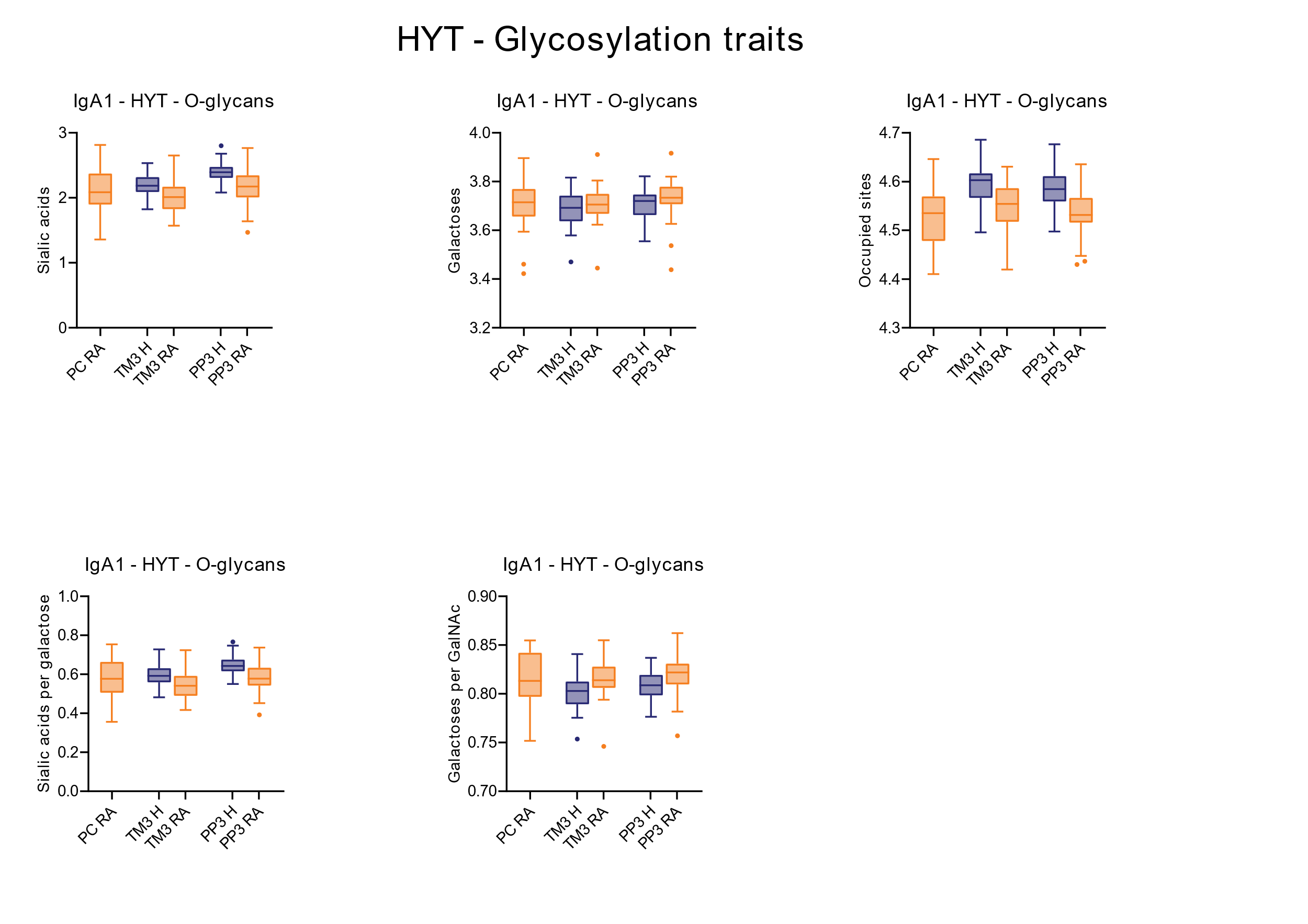

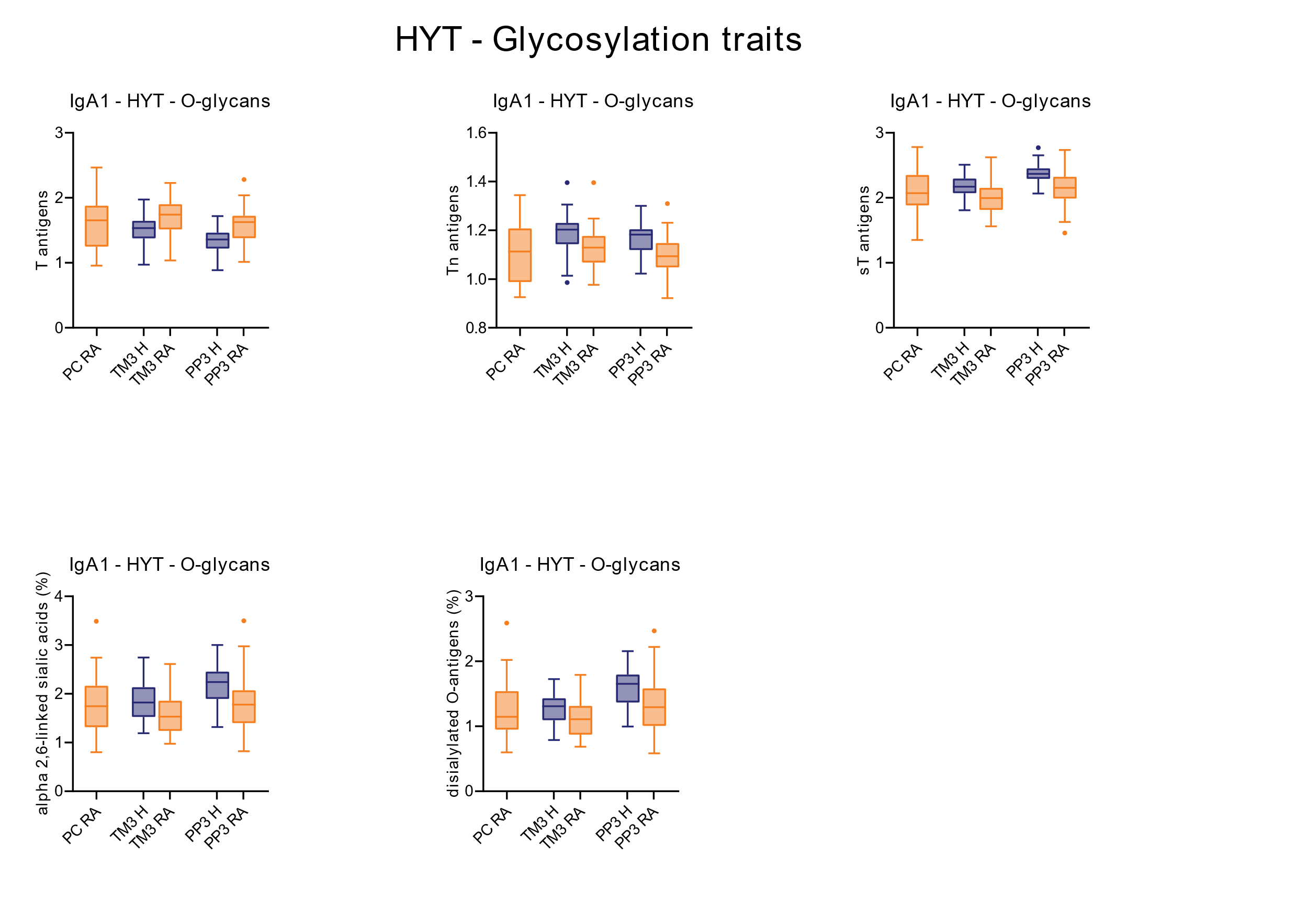

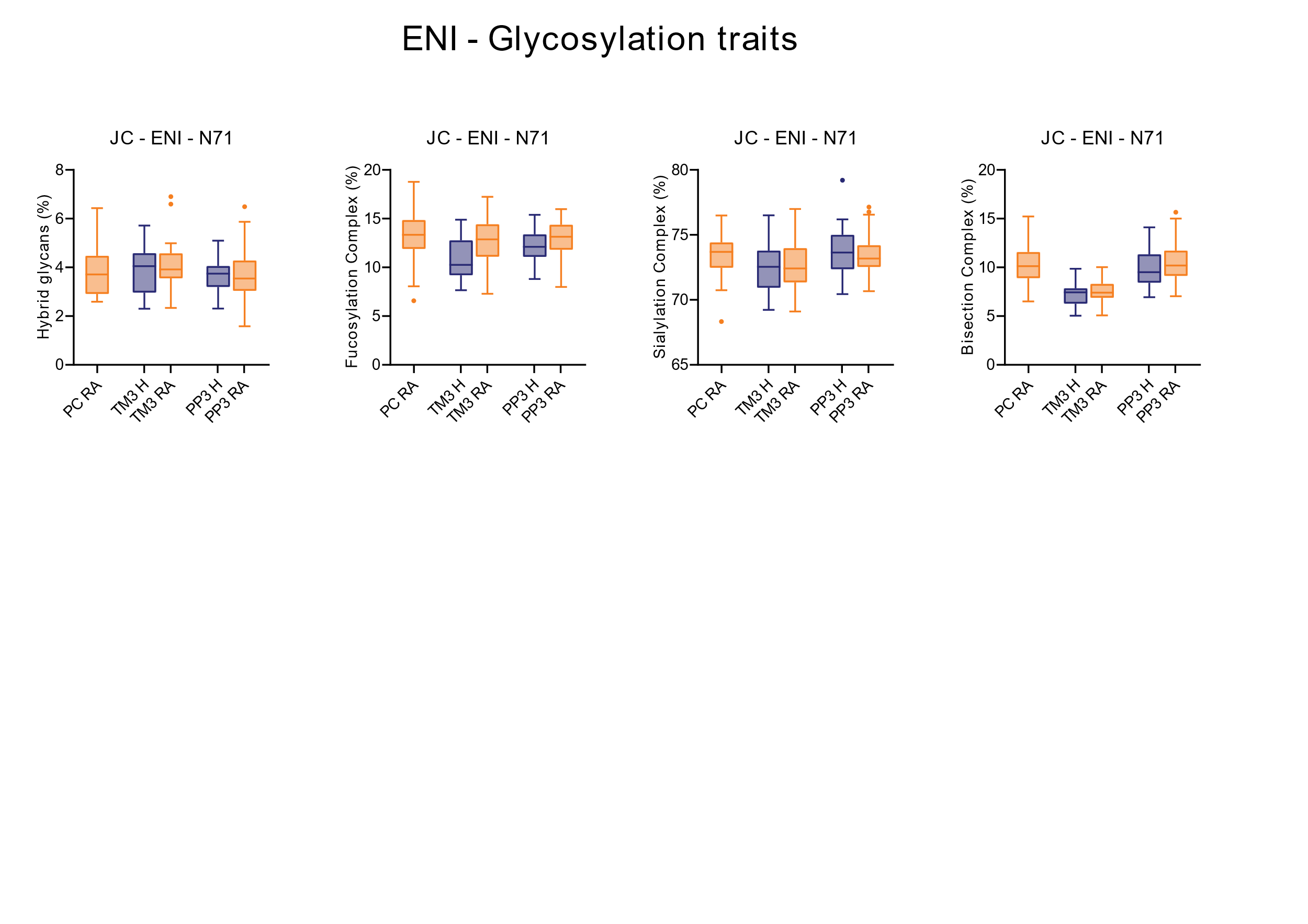

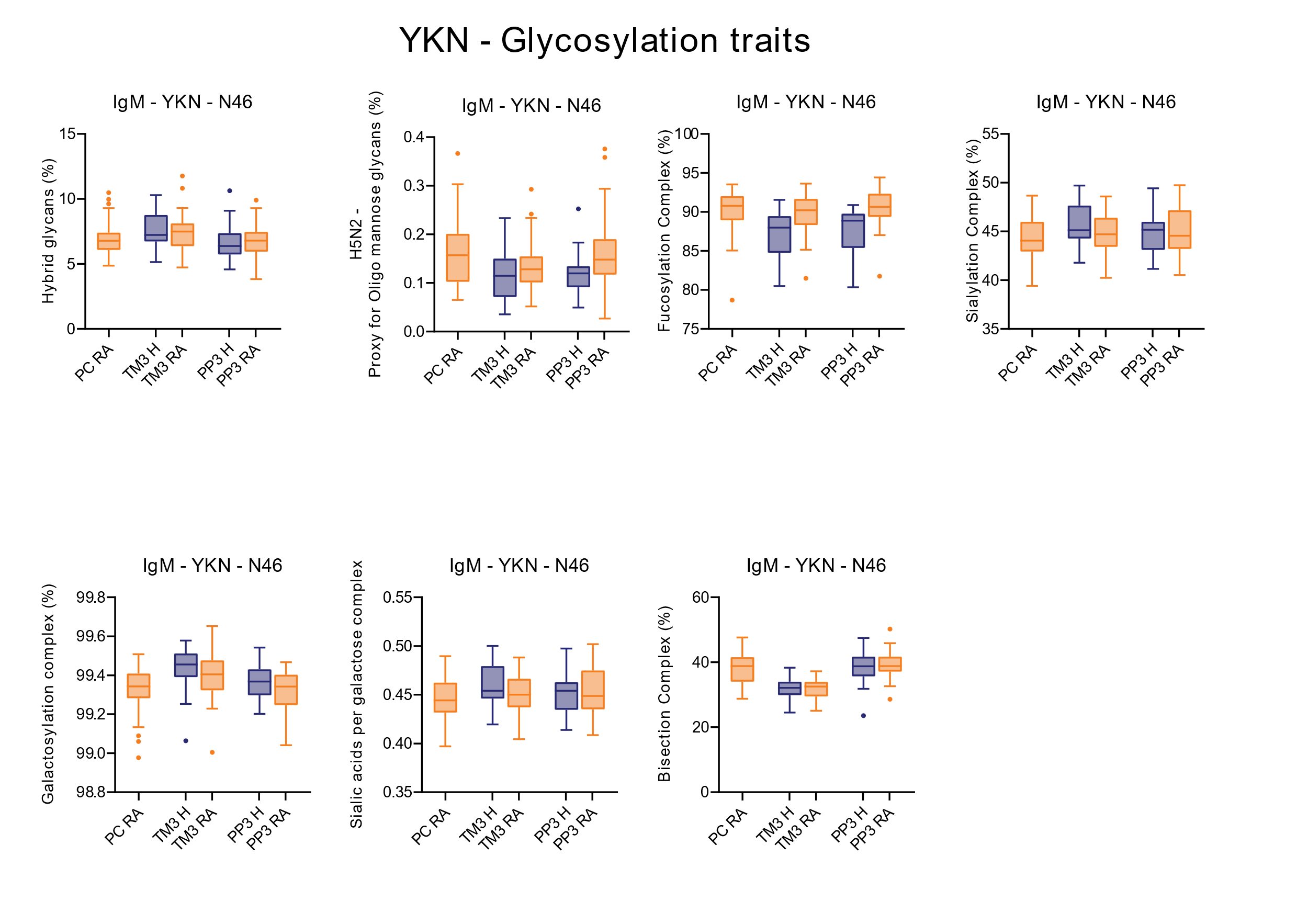

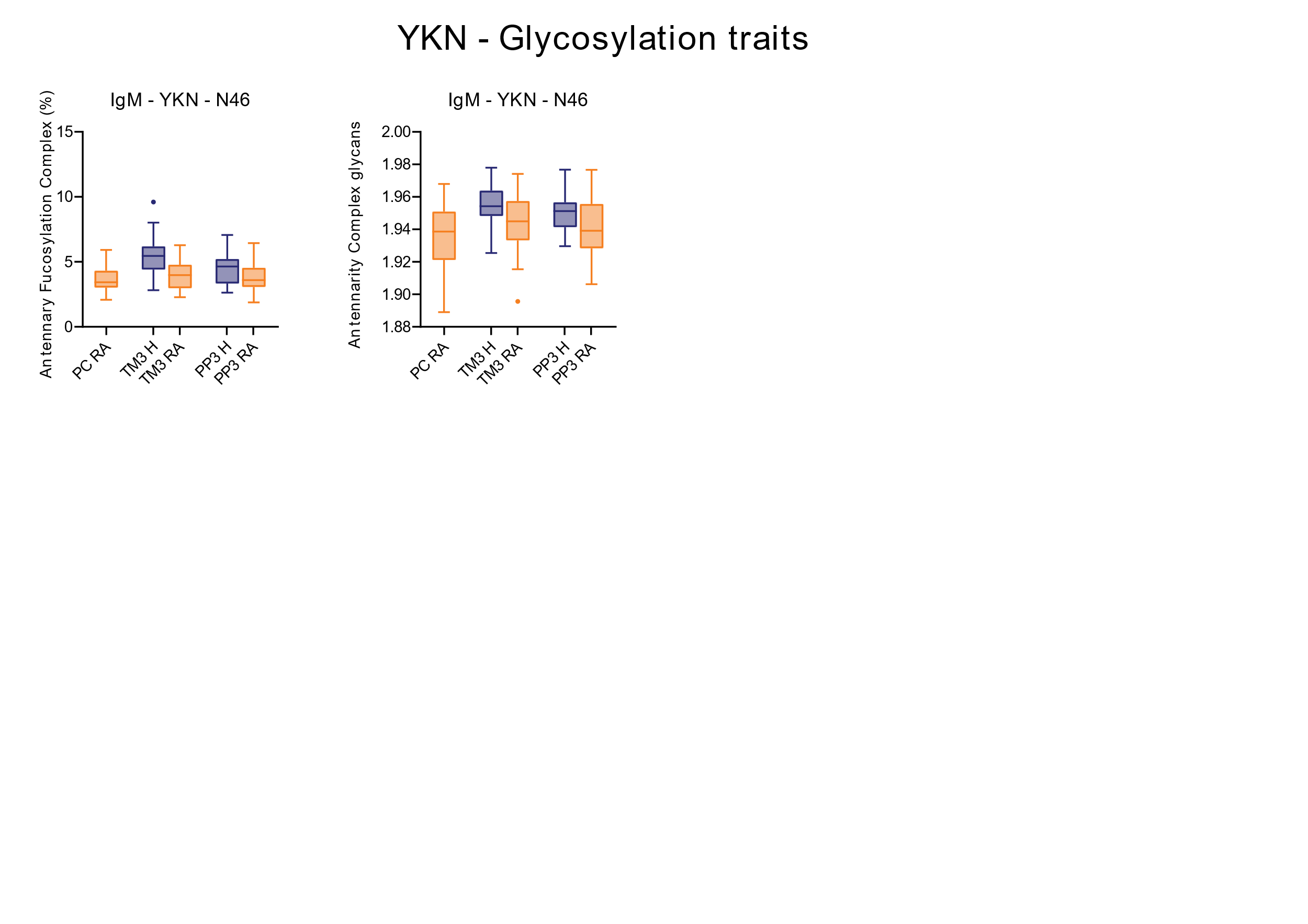

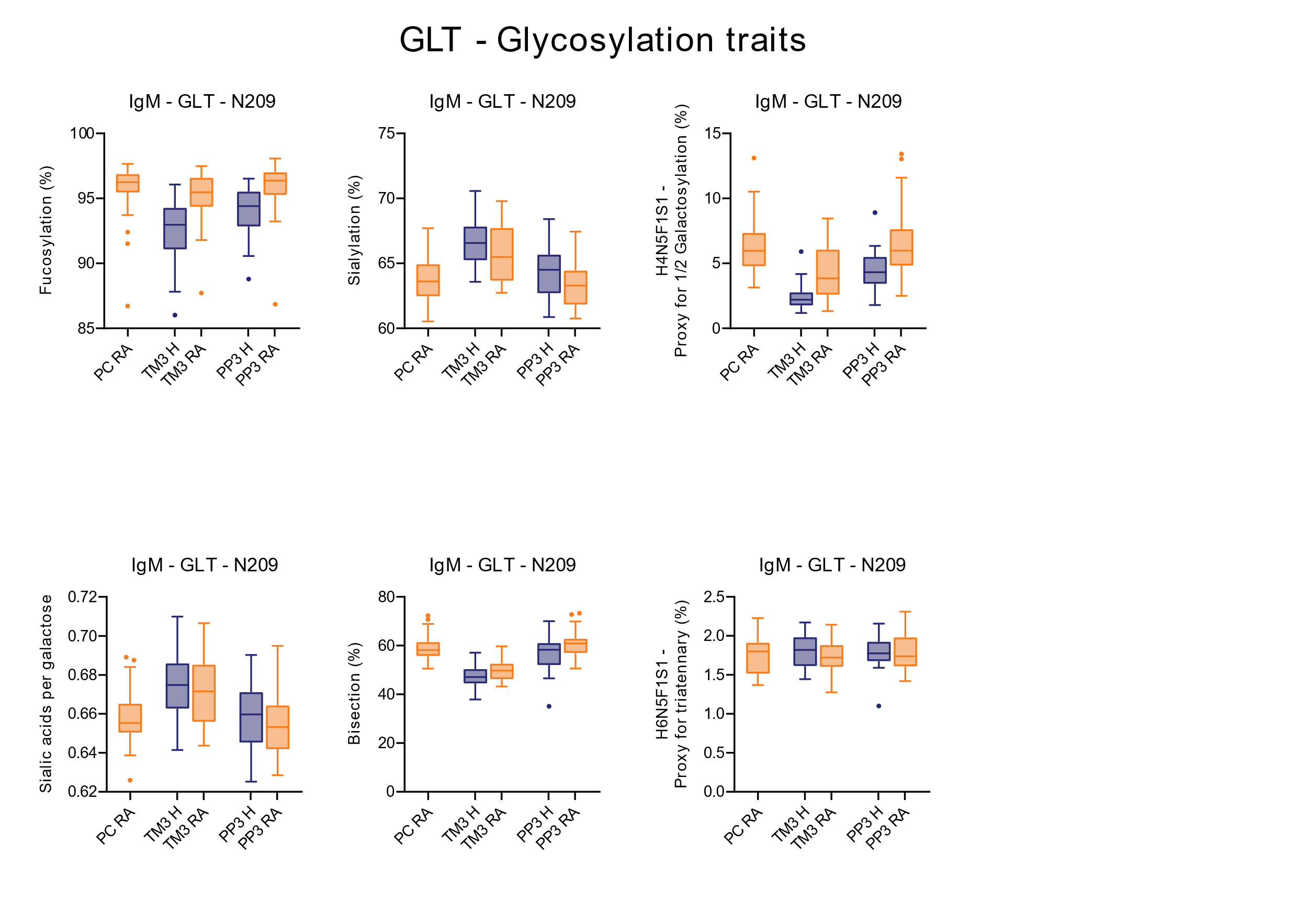

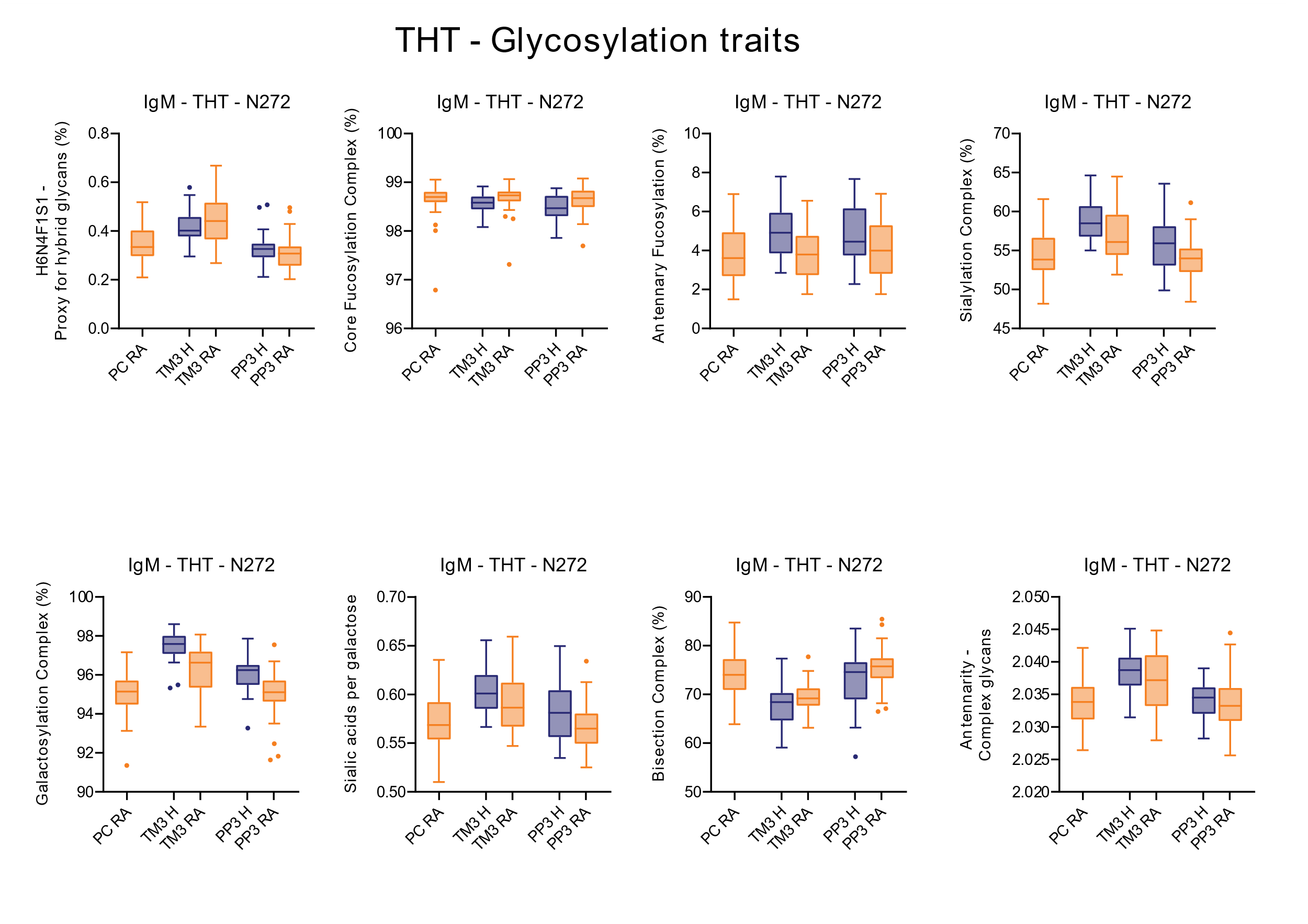

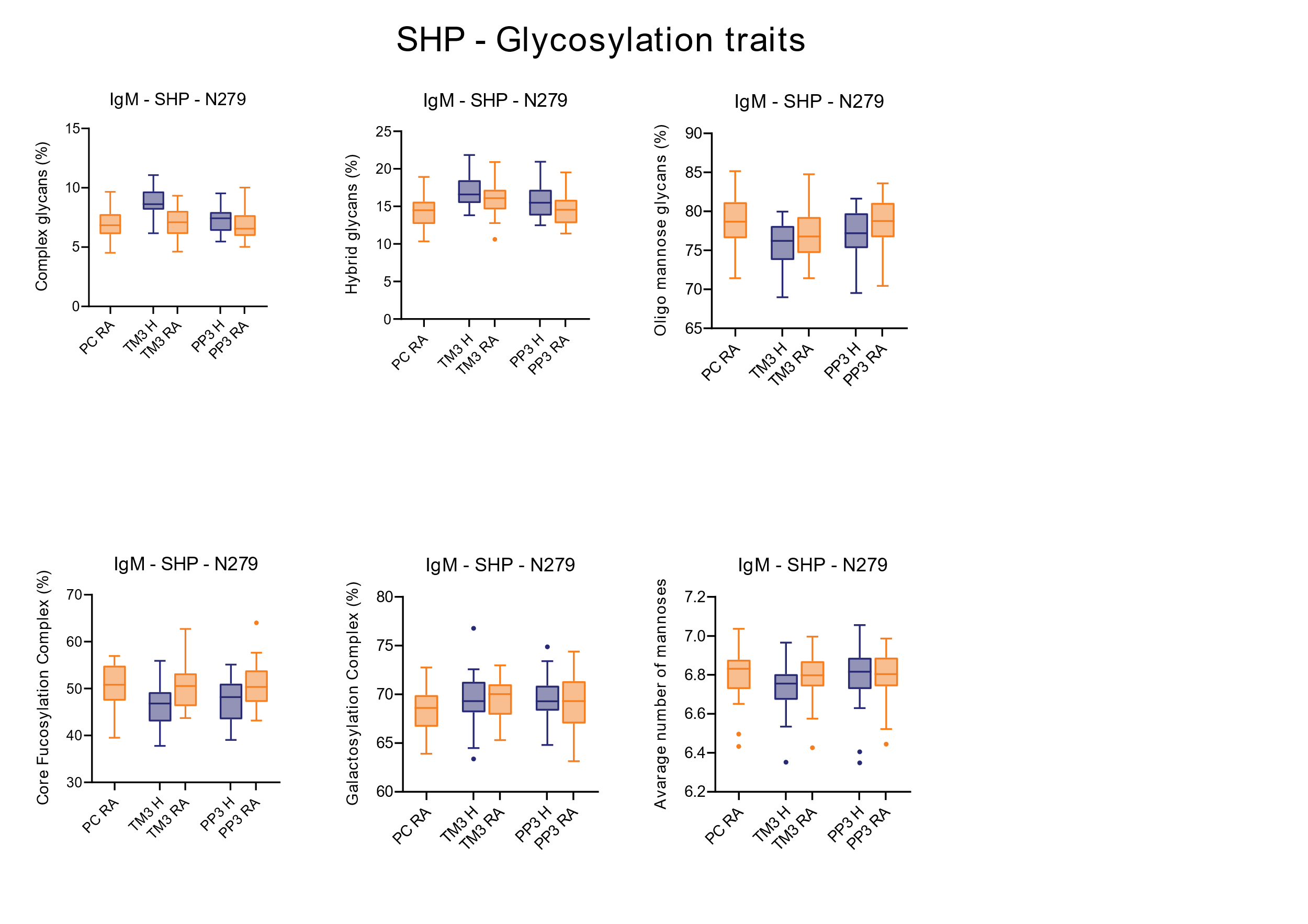

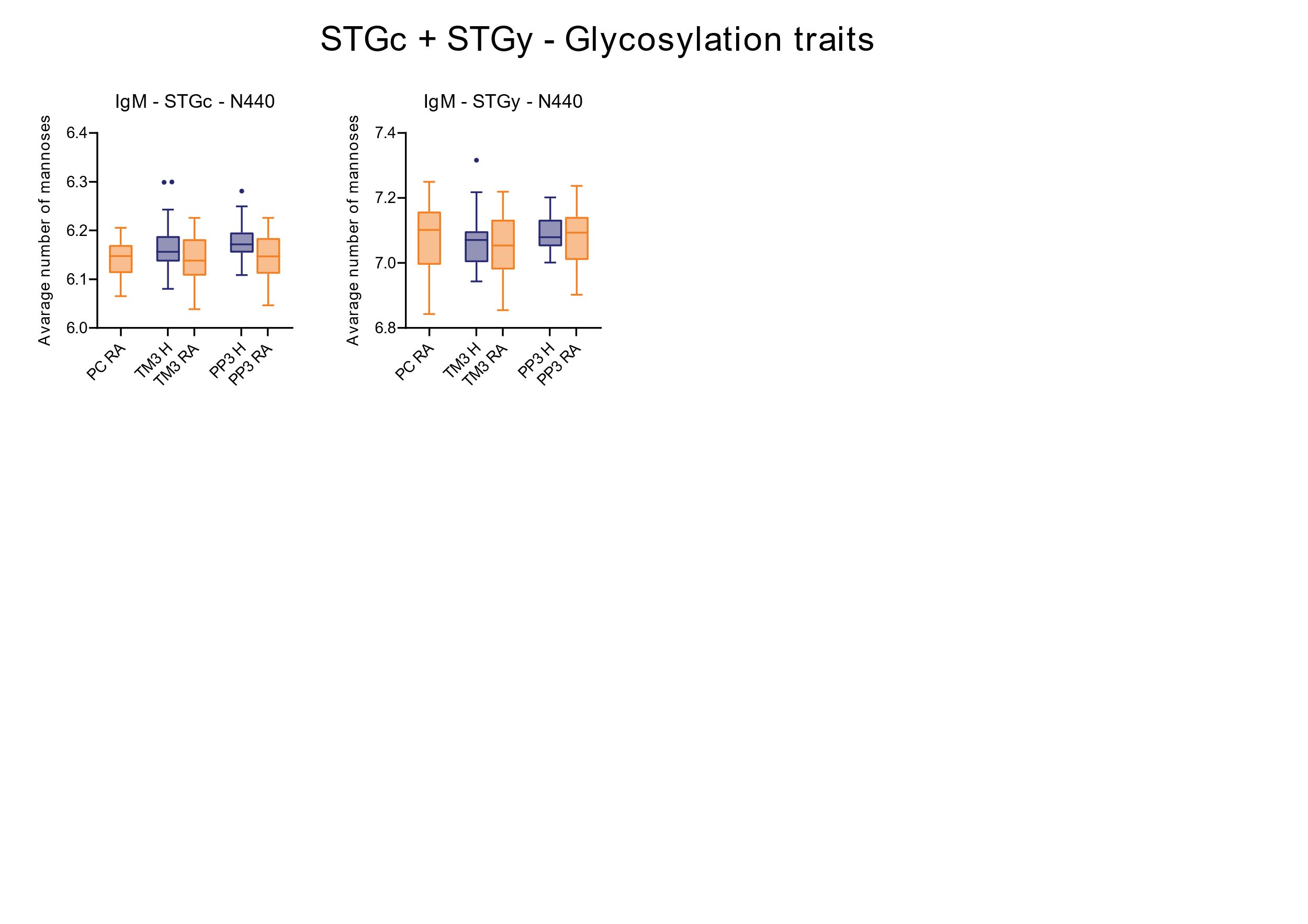


**Figure S5**: Glycosylation changes during pregnancy and associations with rheumatoid arthritis of IgG, IgA, IgM, and JC glycosylation traits.

## Supplementary methods

**Chemicals and enzymes**

| **Compound** | **Abbreviation** | **CAS#** | **Source or reference** |
| --- | --- | --- | --- |
| CaptureSelect™ FcXL Affinity Matrix beads (binding capacity IgG 25−35 mg/mL; 5 mL) | IgG capturing beads | - | Thermo Fisher Scientific, Leiden, The Netherlands # 194328005 |
| CaptureSelect™ IgA Affinity Matrix (binding capacity IgA ~8 mg/mL; 5 mL) | IgA capturing beads | - | Thermo Fisher Scientific # 194288005 |
| POROS™ CaptureSelect™ IgM-XL Affinity Matrix (binding capacity IgM >5 mg/mL; 5 mL) | IgM capturing beads | - | Thermo Fisher Scientific # 2812892005 |
| Sequencing grade modified trypsin | Trypsin |  | Promega, Madison, WI #V5117 |
| Glu-C, Sequencing Grade | Glu-C |  | Promega #V1651 |
| *Chemicals* |  |  |  |
| Sodium phosphate dibasic dihydrate | Na_2_HPO_4_·2 H_2_O | 10028-24-7 | Merck KGaA, Darmstadt, Germany, # 71643-1KG |
| Potassium dihydrogen phosphate | KH_2_PO_4_ | 7778-77-0 | Merck KGaA #1.04873.1000 |
| Sodium Chloride | NaCl | 7647-14-5 | Merck KGaA # 31434 |
| Ammonium bicarbonate | ABC | 1066-33-7 | Merck KGaA # 09830 |
| Formic Acid | FA | 64-18-6 | VWR, #84865.180 |
| tris(2-carboxyethyl)phosphine hydrochloride | TCEP | 51805-45-9 | Merck KGaA # 646547-10X1ML |
| 2-chloracetamide | CAA | 79-07-2 | Merck KGaA #C0267 |
| *Solvents* |  |  |  |
| Acetonitrile | ACN | 75-05-8 | Fluka #34967 |
| trifluoroacetic acid | TFA | 76-05-1 | Fluka #40967 |
| **Solutions** | **Abbreviation** | **Recipe** | |
| Phosphate-buffered saline | PBS | 32 mM sodium phosphate dibasic dihydrate, 3.5 mM potassium dihydrogen phosphate and 145 mM sodium chloride; pH 7.6 | |

Water was purified via a Purelab Ultra, maintained at 18.2Ω (Veolia Water Technologies Netherlands B.V., Ede, NL).

**Serum collection and Storage**

**Glycopeptide analysis by liquid chromatography - mass spectrometry**
Briefly, sample loading was performed using an autosampler equipped with a 2.4 µL needle and 5 µL PEEK sample loop. From each well, 200 nL of sample was loaded in 0.02% TFA onto a Acclaim™ PepMap™ C18 trap column (300 µm x 5 mm, particle size 5 μm, pore size 100 Å, Thermo Fisher Scientific), at a flow of 25 µL/min. After 1 min the trap column was switched in-line with a nanoEase M/Z Peptide BEH C18 analytical column (75 μm x 100 mm, particle size 1.7 μm, pore size 130 Å, 1/PK, Waters) at a flow of 0.6 μL/min. A linear gradient of solvent A (0.02% TFA in water) and solvent B (95% acetonitrile) was applied: 3%B 0 min, 30%B 6.5 min, 95%B 10 min, 95%B 10−12 min, 3%B 13 min, 3%B 13−21 min. Positive-mode ESI was performed using a Captive Spray source equipped with nanoBooster technology (Bruker Daltonics). Ionization was enhanced by enriching the nebulizing nitrogen gas (0.4 bar) with acetonitrile. The collision energy was at 5 eV, the transfer time at 110 μs, and the prepulse storage at 21 μs. The acquisition window was set to m/z 400−1800 at a frequency of 1 Hz.

**GlycoDash processing**

For quality control, analyte presence was evaluated per charge state based on the following quality criteria: Based on isotopic pattern quality score (IPQ <0.2), signal-to-noise ratio (S/N > 9), and absolute mass error (<20 ppm). For each glycopeptide cluster, the total area of all analytes passing the analyte quality criteria was summed over all included charge states. During spectral quality control, data of specific glycopeptide clusters were excluded from further processing per sample when either the respective total intensity or the total number of analytes passing the criteria was in the lower second percentile of all samples. After the exclusion of low-quality clusters, individual analytes were subjected to the analyte criteria described above to determine which were of sufficient quality for relative quantification. Glycopeptides in specific charge states that were of sufficient quality in more than 50% of the samples per group (the five combinations of disease status and time point differentiated in Figure 4) were included for relative quantification. Finally, for analytes that passed quality control, the absolute areas for all included charges per glycopeptide were summed, corrected for the isotopic fraction integrated, and normalized to the total area per glycopeptide cluster, resulting in the relative intensities of the glycopeptide compositions.
